# Shielding Unit Engineering of NIR-II Molecular Fluorophores for Improved Fluorescence Performance and Renal Excretion Ability

**DOI:** 10.3389/fchem.2021.739802

**Published:** 2021-09-02

**Authors:** Chunchen Liu, Huilong Ma, Zhubin Hu, Rui Tian, Rui Ma, Yifan Xu, Xinyuan Wang, Xingfu Zhu, Panpan Yu, Shoujun Zhu, Haitao Sun, Yongye Liang

**Affiliations:** ^1^ Department of Materials Science and Engineering, Shenzhen Key Laboratory of Printed Organic Electronics, Southern University of Science and Technology, Shenzhen, China; ^2^ State Key Laboratory of Precision Spectroscopy, East China Normal University, Shanghai, China; ^3^ State Key Laboratory of Molecular Vaccinology and Molecular Diagnostics and Center for Molecular Imaging and Translational Medicine School of Public Health, Xiamen University, Xiamen, China; ^4^ State Key Laboratory of Supramolecular Structure and Materials, College of Chemistry, Jilin University, Changchun, China

**Keywords:** NIR-II fluorophores, shielding unit, S-D-A-D-S structure, fluorescence performance, renal excretion

## Abstract

Molecular fluorophores emitting in the second near-infrared (NIR-II) window with good renal excretion ability are favorable for *in vivo* bio-imaging and clinical applications. So far, renally excretable fluorophores are still less studied. Understanding the influences of molecular structure on optical properties and renal excretion abilities are vital for fluorophore optimization. Herein, a series of shielding unit-donor-acceptor-donor-shielding unit (S-D-A-D-S) NIR-II molecular fluorophores are designed and synthesized with dialkoxy chains substituted benzene as the S unit. The anchoring positions of dialkoxy chains on benzene are tuned as meso-2,6, para-2,5, or ortho-3,4 to afford three fluorophores: BGM6P, BGP6P and BGO6P, respectively. Experimental and calculation results reveal that alkoxy side chains anchored closer to the conjugated backbone can provide better protection from water molecules and PEG chains, affording higher fluorescence quantum yield (QY) in aqueous solutions. Further, these side chains can enable good encapsulation of backbone, resulting in decreased binding with albumin and improved renal excretion. Thus, fluorophore BGM6P with meso-2,6-dialkoxy chains exhibits the highest quantum yield and fastest renal excretion. This work emphasizes the important roles of side chain patterns on optimizing NIR-II fluorophores with high brightness and renal excretion ability.

## Introduction

To date, remarkable attention has been attracted for developing fluorophores for biological imaging in the second near-infrared window (1,000–1700 nm, NIR-II) due to the merits of deep photon penetration and low noise interference (Hong et al., 2017; Li and Pu, 2019; Lei and Zhang, 2021). Fluorophores based on organic molecules exhibit superiority because of good biocompatibility and easy tunability on optical performance (Antaris et al., 2016; Ding et al., 2018; Kenry et al., 2018). Recently, indocyanine green (ICG) was utilized for the first in-human NIR-II imaging guided liver-tumor surgery (Hu et al., 2020). However, great challenges need to be addressed for clinical administration of most reported NIR-II molecular fluorophores due to their unfavorable metabolic pathway with apparently accumulation in functional tissues and organs (Yang et al., 2017; Tian et al., 2019; Feng et al., 2021). Molecular fluorophores with rapid clearance from body but low retention in tissues and organs are preferred for preclinical studies and clinical translation (Li et al., 2020). Renal excretion can enable fast clearance of fluorophores after intravenous injection treatment (Wan et al., 2018). However, very few NIR-II fluorophores have been reported to be renally excreted, and it still lacks guidance for designing molecular fluorophores with simultaneously improved fluorescence performance and renal excretion efficiency (Huang et al., 2019; Yang et al., 2021). Therefore, it is of great significance to study the relationship between molecular structure and optical properties as well as renal excretion ability of NIR-II fluorophores.

A molecular architecture with shielding unit-donor-acceptor-donor-shielding unit (S-D-A-D-S) backbone was previously proposed by our group to develop water soluble NIR-II molecular fluorophores (Yang et al., 2018; Ma et al., 2020; Yang et al., 2021). The effects of backbone structures and side chains of D units on fluorescence performance of S-D-A-D-S fluorophores have been elucidated (Yang et al., 2018). It was demonstrated that the optimized D units could result in less molecular backbone distortion, weaker intermolecular interaction and enhanced protection of molecular backbone from interaction with water, which would effectively improve the absorption coefficients and quantum yields (QYs) of fluorophores in aqueous solutions (Yang et al., 2018). S units also play an important role on influencing fluorescence performance of the S-D-A-D-S fluorophores. It was showed that altering the side chain length on S units could simultaneously regulate the fluorescence performance and renal excretion efficiency of fluorophores (Wan et al., 2018).

**FIGURE 1:**
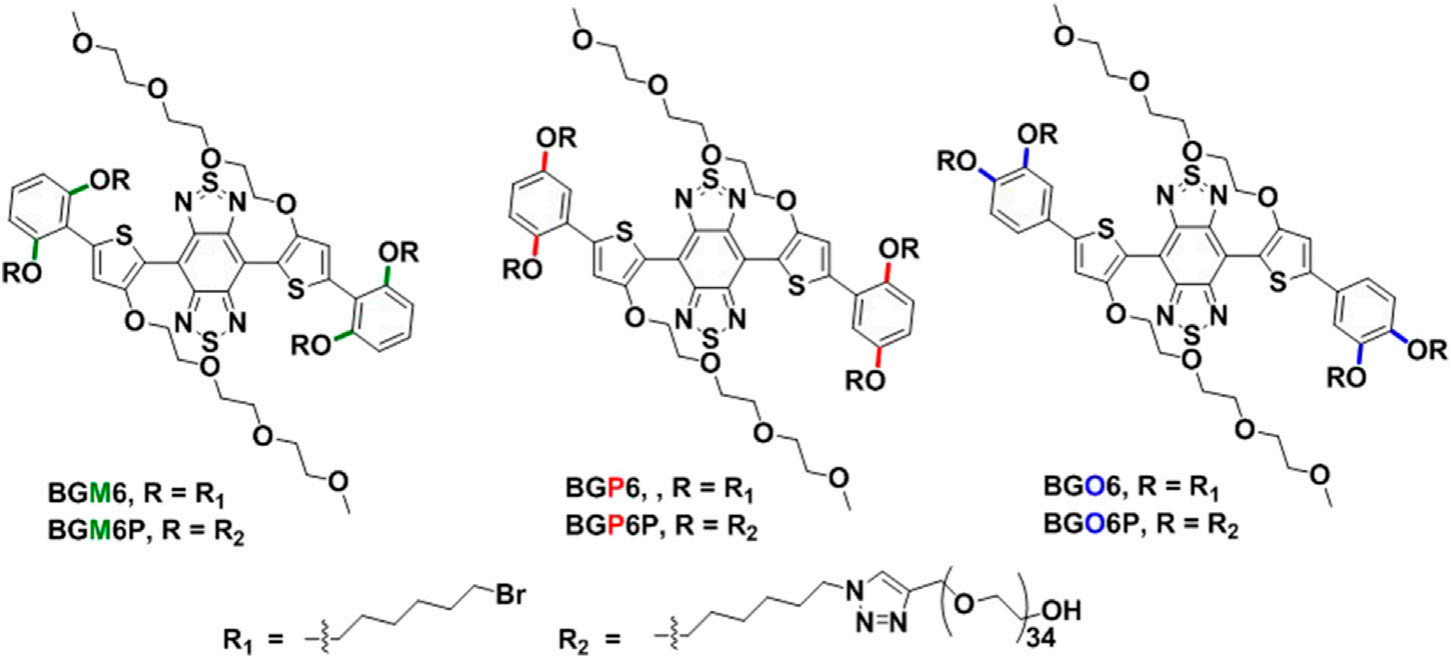
Structures of the S-D-A-D-S molecular fluorophores.

Herein, a series of S-D-A-D-S NIR-II molecules are designed and synthesized with dialkoxy chains substituted benzene as S unit, 3- (2-(2-(2-methyoxyethoxy)ethoxy)ethoxy (TEG) substituted thiophene as D unit and benzo [1,2-c:4,5-c’]bis [1,2,5]thiadiazole (BBTD) as A unit (Figure 1). The anchoring positions of dialkoxy chains on benzene shielding unit are varied as meso-2,6, para-2,5 or ortho-3,4 to afford fluorophores BGM6P, BGP6P and BGO6P, respectively. These fluorophores exhibit NIR-II fluorescence with emission peaks over 1,040 nm. It is found that the closer arrangement of dialkoxy chains in S units to molecular backbone can enable better protection of molecular backbone, leading to higher fluorescence QY. As a result, BGM6P with meso-2,6-dialkoxy chains substituted S unit exhibits the highest QY of 0.12% (with reference to IR-26 with a QY of 0.05% in dichloroethane) in aqueous solutions. Besides, the PEG chains of BGM6P can form better encapsulation of hydrophobic backbone, resulting in its weaker protein binding affinity. Therefore, BGM6P also shows the highest renal clearance efficiency and lowest liver, skin and muscle uptake within the first 6 h post-injection in mice.

## Results and Discussion

### Molecular Design

As illustrated in Figure 1, the conjugated backbone is constructed with dialkoxy chains substituted benzene as S unit, thiophene as D unit and BBTD as A unit because such backbone has been reported to afford NIR-II fluorescent emission and renal excretion ability (Wan et al., 2018). Four polyethylene glycol-1500 (PEG-1500) chains are conjugated on the end of four alkoxy chains *via* click reaction between terminal alkynyl groups of PEG chains and azide groups of alkoxy chains. It is noteworthy that the substitution positions of dihexyloxy chains on S unit are elaborately engineered as meso-2,6, para-2,5 or ortho-3,4 position to yield three molecular fluorophores BGM6P, BGP6P and BGO6P, respectively. It provides a chance to scrutinize the influence of side chains substitution positions of S unit on optical performance and renal excretion behaviors of the S-D-A-D-S molecular fluorophores.

### Optical Properties

**FIGURE 2:**
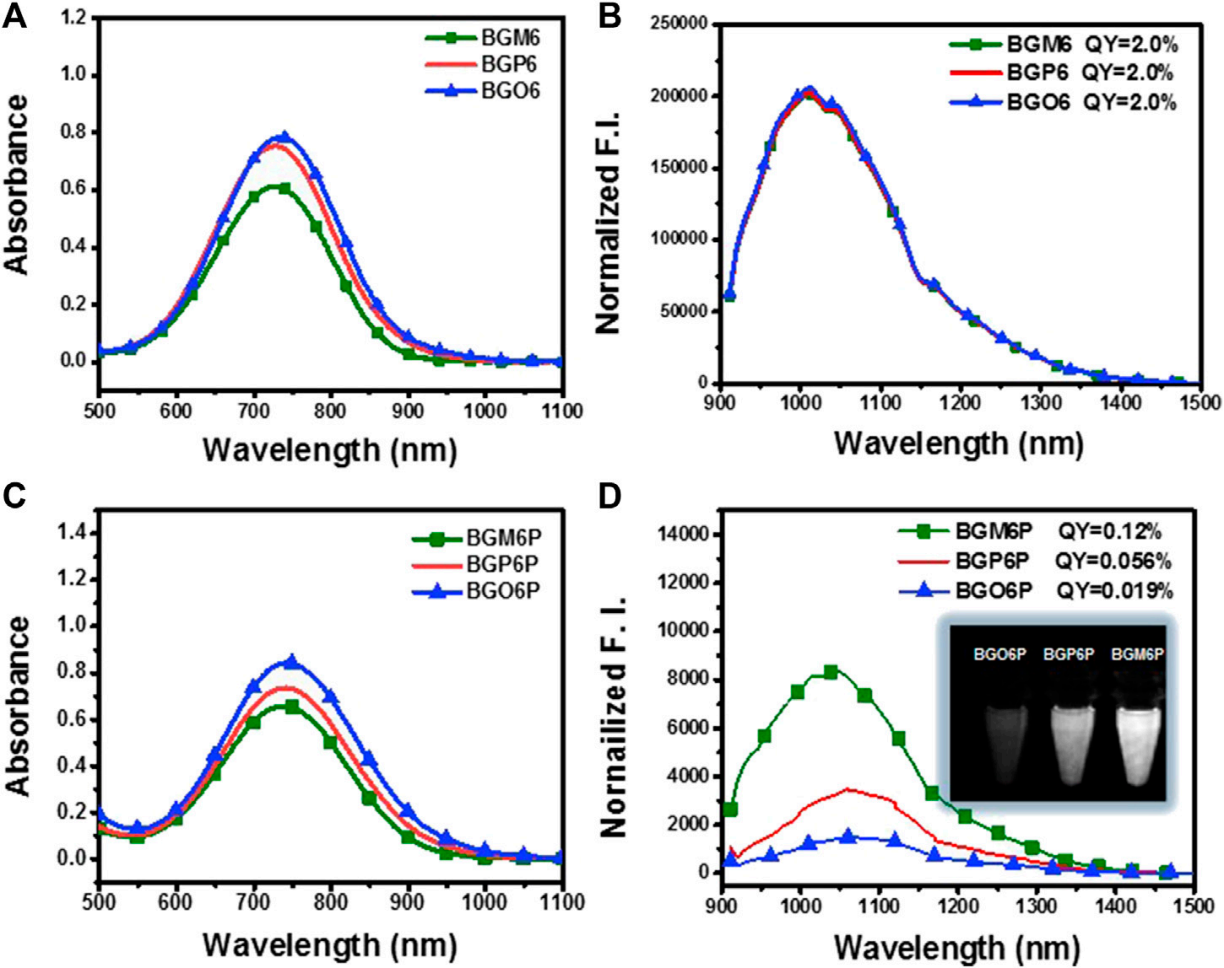
**(A)** Absorption and **(B)** emission spectra of un-PEGylated fluorophores in toluene (40 μM). **(C)** Absorption and **(D)** emission spectra of PEGylated fluorophores in water (80 μM). An 808 nm laser is used for excitation with exposure time of 50 ms. The fluorescence intensity is normalized with the optical density (OD) of the sample at 808 nm. The inset of Figure 2D displays the brightness of fluorophore aqueous solutions under an 808 laser excitation at same concentration.

The influence of dialkoxy substitution positions of S unit on optical properties is investigated (Figures 2A–D and Table 1). In toluene, all un-PEGylated fluorophores show similar absorption spectra with peaks at ∼730 nm, which is consistent with the similar calculated energy gap (Supplementary Figure S2). Both BGO6 and BGP6 show high peak absorption coefficient of around 19 × 10^3^ M^−1^ cm^−1^, while the one of BGM6 is slightly lower. It could be explained by the larger backbone distortion of BGM6 than BGO6 and BGP6 (Supplementary Figure S3). Similar fluorescence spectra with emission maximum located at 1,013 nm are observed for all fluorophores in toluene, and comparable QYs of ∼2.0% (QY of IR-26 with 0.05% in 1,2-dichloroethane as reference) are determined.

**TABLE 1:** Optical data of the fluorophores. QE (brightness) = QY × *ε*.

Fluorophores	Solvent	*ε*_max_ (10^3^ M^−1^ cm^−1^)	*λ*_abs_ (nm)	*λ*_em_ (nm)	Stokes shift (nm)	QY (%)	Qe
BGM6	Toluene	15	728	1,013	285	2.0	300
BGP6	Toluene	19	727	1,013	286	2.0	380
BGO6	Toluene	19	733	1,013	280	2.0	380
BGM6P	Water	8.1	736	1,047	311	0.12	9.72
BGP6P	Water	9.7	736	1,060	324	0.056	5.43
BGO6P	Water	11	741	1,060	319	0.019	2.01

The fluorophores BGO6P, BGP6P and BGM6P with PEG chains exhibit similar absorption spectra in water with peaks at ∼740 nm, slightly red-shifted compared to those in toluene. The peak absorption coefficient of BGM6P (8.1 × 10^3^ M^−1^ cm^−1^) is slightly lower than those of BGO6P and BGP6P (11 × 10^3^ M^−1^ cm^−1^ and 9.7 × 10^3^ M^−1^ cm^−1^) (Figure 2C). All PEGylated fluorophores show similar emission spectra with peaks at 1,047–1,060 nm, which are all redshifted when compared with their counterparts in toluene. Interestingly, the dialkoxy chains anchoring positions on S unit makes a distinct difference on the QYs of fluorophores in water. The highest QY of 0.12% is measured for BGM6P, outperforming 0.056% of BGP6P and 0.019% of BGO6P. Consequently, BGM6P aqueous solution displays the highest brightness of 9.72 at the same concentration under an 808 nm laser excitation, higher than 5.43 of BGP6P and 2.01 of BGO6P (Figure 2D), suggesting its superiority for applications on *in vivo* biological imaging. Large Stokes shifts of over 310 nm can be observed for all fluorophores in water, which can prevent the self-absorption of fluorescence and thus further improve biological imaging quality.

### Molecular Dynamics Simulation in the Aqueous Environment

**FIGURE 3:**
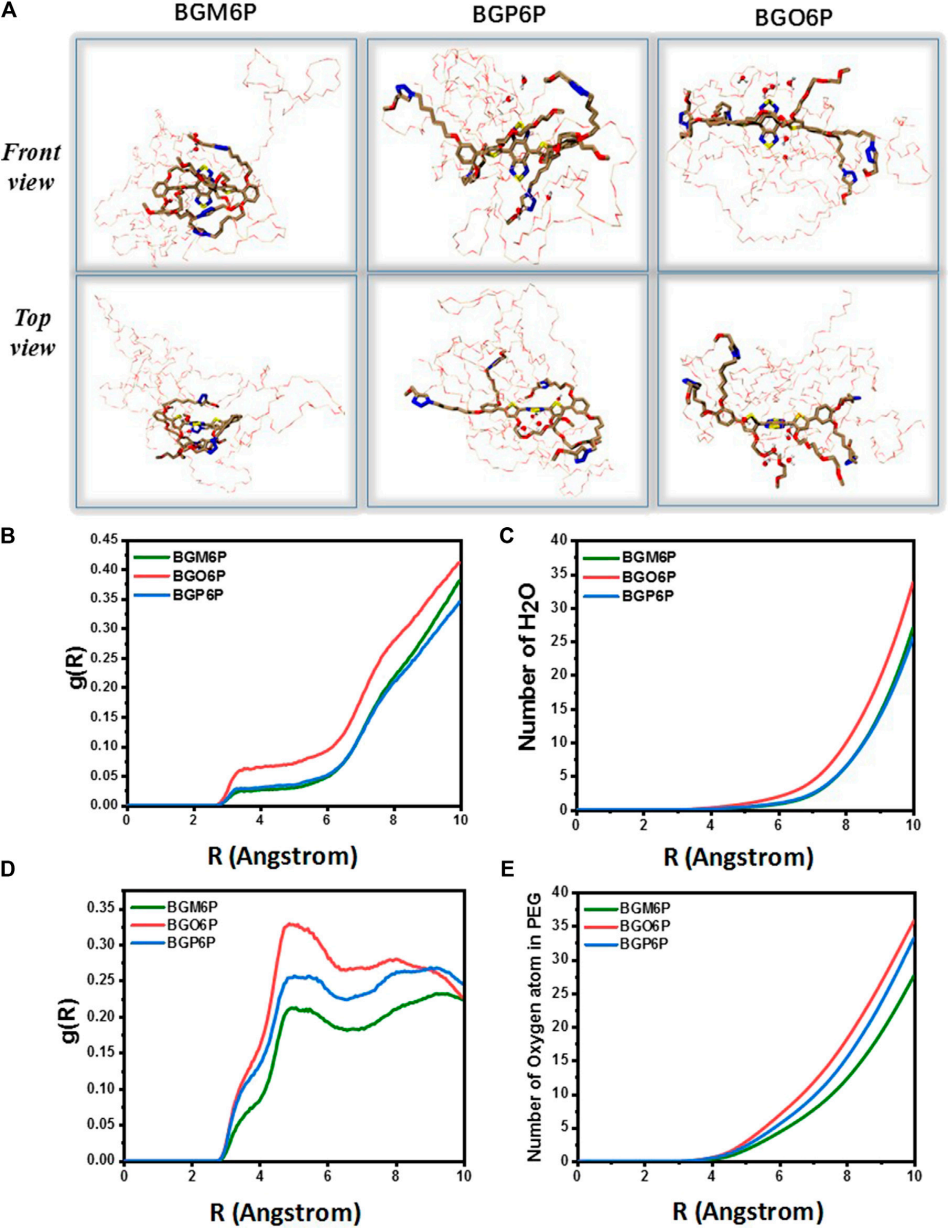
**(A)** The front view **(up)** and top view **(below)** of fluorophores in aqueous environment from molecular dynamics (MD) simulations. PEG chain: the bray and red thin part; carbon atom: brown; oxygen atom: red; sulfur atom: yellow; nitrogen: blue. **(B)** Radial distribution function (RDF) of oxygen atoms in water molecules and **(C)** counted the number of water molecules around the BBTD unit of fluorophores. **(D)** Radial distribution function (RDF) of oxygen atoms in PEG chains and **(E)** counted umber of oxygen atoms in PEG chains around the BBTD unit of fluorophores, where g(R) represents RDF **(left)**, R(Å) means the radius in angstrom.

Molecular dynamics (MD) simulations are conducted to reveal the interactions between fluorophores and surrounding water molecules (Yang et al., 2018; Ma et al., 2020; Zhu et al., 2020). As shown in Figure 3A, four meso-2,6 alkoxy chains on two S units of BGM6P are all extended toward backbone center and thus can effectively cover both thiophene D units and BBTD unit. For BGP6P, it is found that two 2-alkoxy chains are extended toward backbone center while two 5-alkoxy chains are extended away from the molecular backbone, affording half coverage effect on thiophene and BBTD units. By contrast, ortho-3,4 dialkoxy chains are all extended away from the molecular backbone center for BGO6P, leading to absence of coverage on thiophene and BBTD units. Radial distribution function (RDF) of oxygen atoms in water molecules and counted number of water molecules (defining the BBTD acceptor unit as the center) are also evaluated (Figures 3B,C). As expected, obviously higher RDF values and more counted number of water molecules surrounding BBTD center can be observed for BGO6P than BGM6P and BGP6P. Considering the fluorescence performance in water can be suppressed with enhanced interaction between molecular backbone and water molecules, the poor protection of ortho-3,4-dialkoxy chains on molecular backbone results in the lowest QY of BGO6P. It should be pointed out that both BGM6P and BGP6P show similar RDF values and counted numbers of water molecules surrounding BBTD center, however, their QYs are different (0.12% for BGM6P while 0.056% for BGP6P). Due to the flexibility of long PEG chains, the twining PEG chains will interact with molecular backbones and decrease the fluorescence performance of fluorophores. Hence, RDF of oxygen atoms in four PEG chains and counted oxygen atoms number (defining the BBTD unit as the center) are calculated (Figures 3D,E). It is revealed that BGM6P exhibits the lowest oxygen atom RDF value and the least counted number of oxygen atoms surrounding BBTD unit. It suggests the weakest interaction between PEG chains and molecular backbone of BGM6P, resulting in the highest QY in aqueous solutions.

### Excretion Behaviors of Fluorophores

**FIGURE 4:**
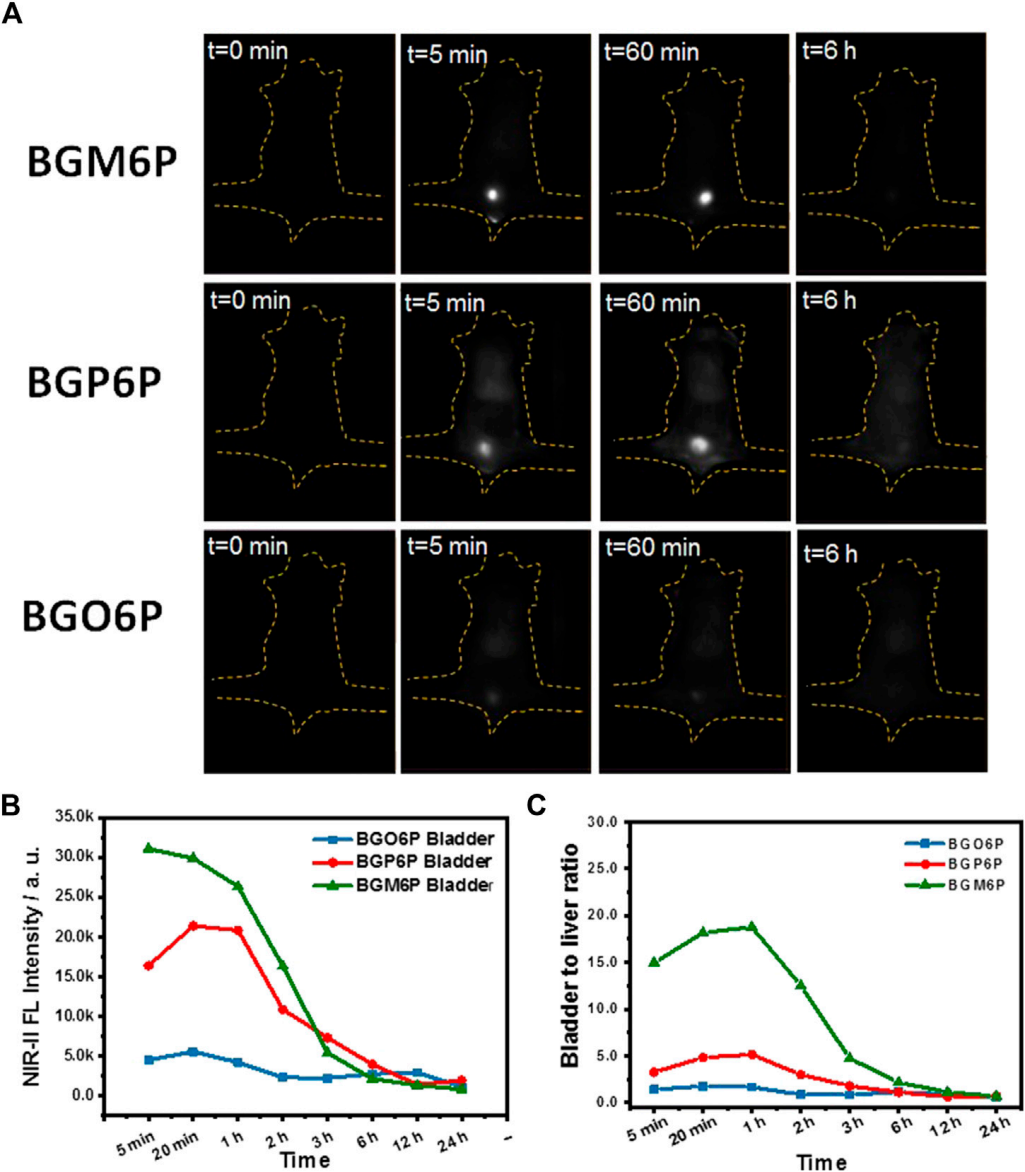
**(A)** Wide-field fluorescence imaging of mice injected with BGM6P, BGP6P and BGO6P as function of time. **(B)** The real-time NIR-II fluorescence intensity in bladder for BGM6P, BGP6P and BGO6P, respectively **(C)** Representative background subtracted signal of bladder to liver as a function of time for mice injected with BGM6P, BGP6P and BGO6P, respectively. All the fluorescence imaging was taken in the NIR-II window (imaging condition: 808 nm laser excitation, power density: 60 mW/cm^2^, exposure time: 100 ms, 1,100 nm long pass (LP) filter, injection dose: optical density (OD) value = 3.4, 200 µL).

Excretion behaviors of BGM6P, BGP6P and BGO6P were investigated to study the influence of molecular structure on renal excretion ability of fluorophores (Figures 4A–C). After intravenous administration of these fluorophores in phosphate buffer saline (PBS) solutions into mice, the real-time distribution of fluorophores was monitored to assess their metabolic progress. Renal excretion pathway can be obviously observed for all three fluorophores, though renal excretion efficiency is different. As shown in Figures 4A,B, for BGM6P treated mouse, the fluorescence signal of bladder reaches the peak value at 5 min post-injection (p. i.), while fluorescence in liver, skin and muscle is almost undetectable. The fluorescence signal in bladder disappears at 6 h p. i. Strong fluorescence signal can also be observed in bladder for BGP6P treated mouse and reaches the peak value at 20 min p. i. and disappears at 12 h p. i. However, conspicuous fluorescence can be figured out in liver, skin and muscle and remained detectable even at 24 h p. i. Presumably due to the inferior fluorescence performance of BGO6P, weak fluorescence signal can be observed in bladder and reaches the peak value at 20 min p. i. and disappears at 12 h p. i. The fluorescence signal in skin and muscle can also be observed at 24 h after intravenous injection. The fluorescence intensity ratio of bladder to liver/skin and muscle are characterized for these three fluorophores and demonstrated that mouse with treatment of BGM6P shows the highest fluorescence ratio of bladder to liver/skin and muscle at any time point after injection (Figure 4C). These results strongly demonstrate that BGM6P with meso-2,6 dialkoxy chains substituted S units exhibits the most effective renal excretion and lowest liver/skin and muscle uptake. The 3-(4,5-dimethylthiazol-2-yl)-2,5-diphenyltetrazolium bromide (MTT) assay was used to evaluate the *in vitro* cytotoxicity of the fluorophores. It is found that more than 95% of cell lines stay alive after incubating with BGM6P (up to 0.1 mg ml^−1^) for 12 h (Supplementary Figure S7), indicating low cytotoxicity of the fluorophore. The biodistribution of BGM6P was measured within 2 h p. i. in mouse (Supplementary Figure S8). The result reveals that there are very weak NIR-II fluorescent signals in all tested main organs, indicating the fast excretion feature of BGM6P.

### Excretion Mechanism of Fluorophores

**FIGURE 5:**
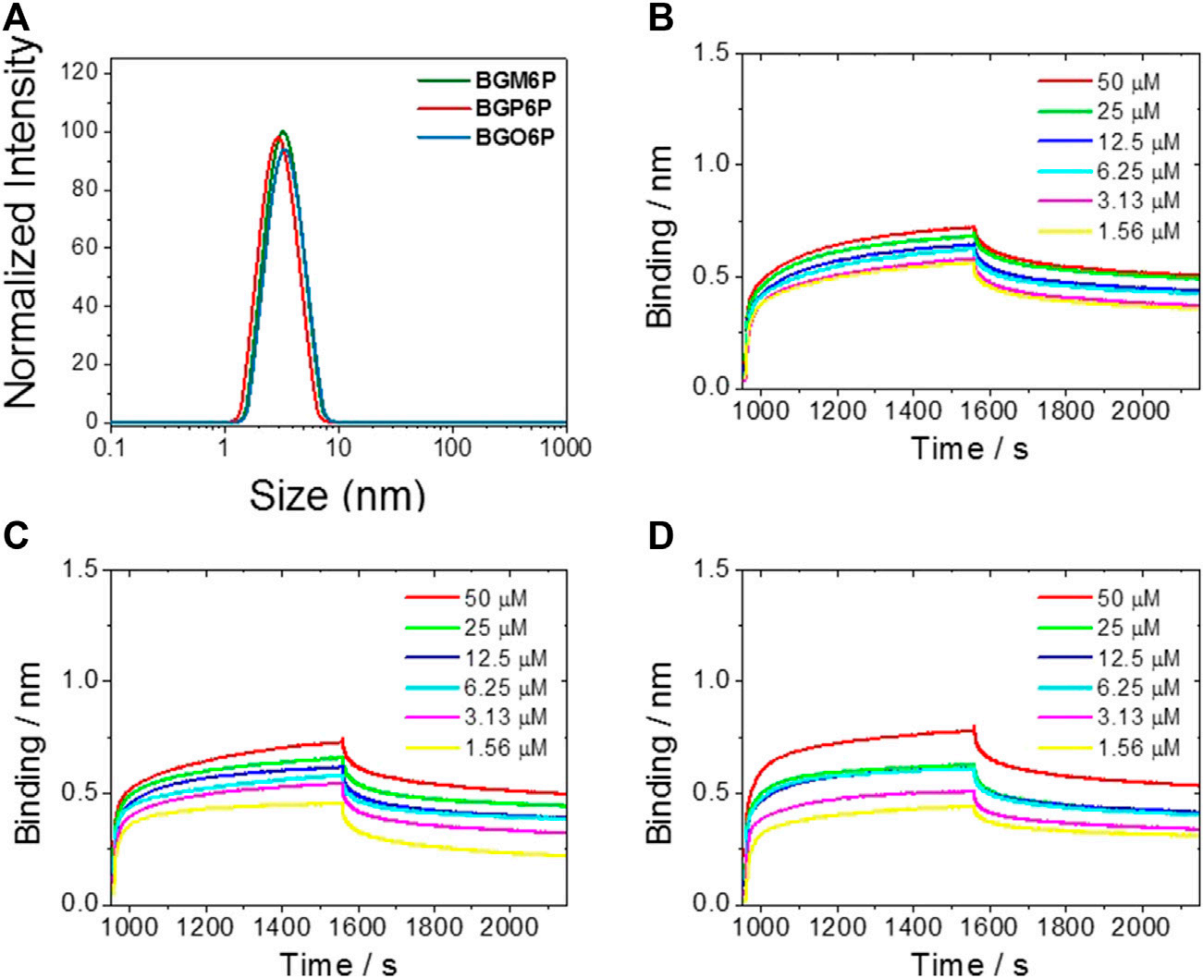
**(A)** Size of fluorophores tested by dynamic light scattering (DLS) method. Kinetic binding assay to albumin results of BGM6P **(B)**, BGP6P **(C)** and BGO6P **(D)** measured by bio-layer interferometry.

After observing that BGM6P exhibits the most effective renal clearance and lowest liver, skin and muscle retention than the other two fluorophores, we attempted to elucidate the relationship between the molecular structure and renal excretion efficiency of these fluorophores. In previous reports, it has been found that the size, hydrophilicity and protein binding affinity of fluorophores are closely related to their excretion ability (Tian et al., 2019). By measuring the size of these fluorophores through the dynamic light scattering (DLS) method (Figure 5A), it is observed that all fluorophores show similar hydrodynamic size of ∼3 nm (Table 2), smaller than the renal cutoff (∼5 nm), which is generally considered as an important requirement for renal excretion. In order to identify the underlying mechanism for the higher renal clearance ability of BGM6P than BGP6P and BGO6P, it should be noted that all meso-2,6 dialkoxy chains and backbone of BGM6P are more perfectly encapsulated by the winding PEG chains than those of BGP6P and BGO6P (Figure 3A). It can afford lower lipophilicity for BGM6P than the other two fluorophores. In this regard, the binding affinities with albumin (the most abundant protein in plasma) of three fluorophores were tested (Figures 5B–D), the dissociation constant (*K*_d_) values are measured to be ∼1.20 μM, ∼0.76 and ∼0.44 μM for BGM6P, BGP6P and BGO6P, respectively (Table 2). The higher *K*_d_ value of BGM6P suggests that it is more difficult for BGM6P to bind with protein, thus leading to its superior renal excretion efficiency to the other two fluorophores.

**TABLE 2:** Size distribution of fluorophores in water (0.05 mg/ml) by dynamic light scattering (DLS) method and kinetic binding assay of fluorophores to albumin measured by bio-layer interferometry.

Fluorophores	Size		Protein binding
		Mean hydrodynamic diameter (nm)	PDI	*K*_d_ (µM)
BGM6P	3.3 ± 0.24	0.236 ± 0.024	1.20 ± 0.27
BGP6P	2.9 ± 0.41	0.210 ± 0.013	0.76 ± 0.17
BGO6P	3.4 ± 0.26	0.228 ± 0.031	0.44 ± 0.13

## Conclusion

In summary, the influence of dialkoxy chains anchoring positions of S units on optical and renal excretion ability of S-D-A-D-S NIR-II molecular fluorophores has been investigated. It is found that meso-2,6 substitutions make the dialkoxy chains arranged closer to molecular backbone than para-2,5 and ortho-3,4 positions. This close arrangement can form favorable protection of molecular backbone from interactions with water as well as PEG chains, thus affording higher fluorescence performance for fluorophore BGM6P. Interestingly, meso-2,6 anchored dialkoxy chains on the S units can also make the backbone better encapsulated by flexible PEG chains than para-2,5 and ortho-3,4 dialkoxy chains, affording weaker protein binding affinity for BGM6P. Consequently, BGM6P shows the fastest renal excretion and lowest liver, skin and muscle accumulation when intravenously injected in mice. This study provides guidelines for the design of NIR-II fluorophores with enhanced fluorescence performance and renal clearance efficiency. It also reveals the importance of tuning side chain substitution positions for optimizing fluorophore design.

## Materials and Methods

### Materials

Unless otherwise noted, all chemical reagents are obtained commercially and utilized without further purification. Tetrahydrofuran (THF), toluene, and dimethyl formamide (DMF) used for reactions were purified by a solvent purification system (Innovative Technology, Inc.) before using. All air and moisture sensitive reactions were carried out in flame-dried glasswares under a nitrogen atmosphere.

### Methods

#### General Measurements

^1^H and ^13^C NMR spectra were performed on 500 and 400 MHz NMR spectrometers (Bruker AVANCE) using CDCl_3_. Mass spectra were recorded on a Q-STAR Elite (ABI) instrument. Ultraviolet-visible-near infrared (UV-Vis-NIR) absorption spectra were recorded on Shimadzu UV-3600PLus. All UV-Vis-NIR measurements were conducted using quartz cuvettes with 1 cm light path and the sample volume was 3 ml. Background measurement was made by using deionized toluene or water without any sample. Size exclusion chromatography (SEC) was performed on Malvern Viscotek 270 max with 10 µm PLgel 600 × 7.5 mm column. THF was used as the mobile phase at a flow rate of 1.0 ml/min at 40°C.

#### Animal Experiments

Animal experiments were approved by the Institute of Radiation Medicine, Chinese Academy of Medical Sciences administrative panel on Laboratory Animal Care. All experiments were performed in accordance with the National Institutes of Health Guide for the Care and Use of Laboratory Animals. C57BL/6 and nude mice were purchased from Charles River. Bedding, nesting materials, food and water were provided ad libitum. Ambient temperature was controlled at 20–22°C with 12-h light/12-h dark cycles.

#### *In vivo* NIR-II Fluorescence Imaging

For dynamic NIR-II imaging, fluorophores were injected from tail vein, injection dose: OD = 3.4, 200 µL. NIR-II fluorescence images were collected using a liquid-nitrogen-cooled, two-dimensional InGaAs array (Princeton Instruments, 640 × 512 pixels). The excitation light was provided by a fiber-coupled 808-nm diode laser (RMPC) with an in-plane excitation power density of 60 mW/cm^2^. The light was collimated and filtered through a 4.5 mm collimator and an 850-nm and a 1000-nm short pass filter (Thorlabs). The emission light was filtered using a 1100-nm, long pass filter (Thorlabs), and focused onto the detector.

## Data Availability

The original contributions presented in the study are included in the article/Supplementary Material, further inquiries can be directed to the corresponding authors.
