# Supplementary material for: Shielding Unit Engineering of NIR-II Molecular Fluorophores for Improved Fluorescence Performance and Renal Excretion Ability
Source: Front Chem. 2021 Sep 2;9:739802. doi: 10.3389/fchem.2021.739802 (PMC8443785; doi:10.3389/fchem.2021.739802)
Supplement: Supplementary file 1 [file DataSheet1.PDF]

Supplementary Material

1 Supplementary Data

1.1 Quantum Yield Measurement

The fluorescence quantum yields of the fluorophores were measured with a similar method to previously reported (Yang et al., 2017; Yang et al., 2018; Ma et al., 2020). The fluorescence spectra in the region of 900-1500 nm were measured by a spectrometer with a thermoelectrically cooled InGaAs detector (HORIBA Ihr320) under an 808 nm diode laser excitation (RMPC lasers, 180 mW). During emission measurements, one 850-nm short pass filter (Thorlabs) was used as the emission filter. The obtained emission spectra were further corrected by the detector sensitivity profile and the absorbance features of the filter. The fluorescence quantum yield was determined against the reference fluorophore **IR-FE** with a known quantum yield of 3.1% (Φ_{st}) in toluene, which was previously determined with **IR-26** of 0.050% as reference in dichloroethane. All samples were measured at 25 ^{o}C with optical density (OD) below 0.1 at 808 nm. The intensity read out from the InGaAs camera was a spectrally integrated total emission intensity in the 900-1400 nm region. Using the measured optical density (OD) at 808 nm and spectrally integrated fluorescence intensity (F), the quantum yield of the test sample can be calculated according to the following equation:

$$\Phi_x(\lambda) = \Phi_{st}(\lambda) \times \frac{F_x}{F_{st}} \times \frac{A_{st}(\lambda)}{A_x(\lambda)} \times \frac{\eta_x^2}{\eta_{st}^2} = \Phi_{st}(\lambda) \times \frac{F_x}{F_{st}} \times \frac{1 - 10^{-OD_{st}(\lambda)}}{1 - 10^{-OD_x(\lambda)}} \times \frac{\eta_x^2}{\eta_{st}^2}$$

Φ_{st} and F_{st} are data of the **IR-FE** standard, Φ_x and F_x are data of the studied sample. η is the refractive index of solvent.

1.2 Density Functional Theory Calculations

To reduce the computational cost, side chains on the benzene units are replaced by methyloxy groups. The ground-state (S_0) geometries of structure-simplified **BGM6**, **BGP6** and **BGO6** were firstly optimized at the B3LYP/6-31G(d) level (Lee et al., 1988; Heyd et al., 2003) and then re-optimized at the tuned-ωB97XD*/6-31G(d) level. The corresponding range separation parameter (ω, in Bohr^{-1}) for each molecule was optimally tuned and listed in **Supplementary Table 1**. The excited-state (S_1) geometries of these molecules were optimized using the time dependent (TD)-tuned-ωB97XD*/6-31G(d) method (Runge and Gross, 1984). The HOMO and LUMO orbitals, absorption excitation

energies of these molecules were obtained at the TD-tuned- ωB97XD*/6-31G(d) level based on their optimized S_0 geometries. The emission excitation energies of these molecules were calculated at the TD-tuned-ωB97XD*/6-31G(d) level based on their optimized S_1 geometries. The polarizable continuum model (PCM) (Tomasi et al., 2005) was employed to take into account the effects of the solvents. All the calculations were performed using the Gaussian 16 software.

1.3 Molecular Dynamic Simulations

For each molecule, the structure was optimized at the PCM(water)-B3LYP/6-311G** level (Lee et al., 1988), and then restrained electrostatic potential (RESP) charges (Bayly et al., 1993) and the General Amber Force Field (GAFF) (Wang et al., 2004) were assigned for the optimized structure. To remove bad contacts before the simulation, 2000 steps of steepest descent followed by 8000 steps of conjugate gradient energy minimizations were carried out. All bonds with hydrogen atoms were fixed using the SHAKE algorithm (Ryckaert et al., 1977). The particle mesh Ewald method with an 8 Å cutoff in real space was used to calculate electrostatic interaction. A Langevin thermostat with a collision frequency of 1.0 ps^{-1} was used to regulate temperature. Isotropic pressure coupling with a relaxation time of 2 ps was used to maintain the pressure to 1 atm. All the MD simulations were performed by AMBER 18 program. The water molecules around the BBTD acceptor center in the effective contact distance (R = 6 Å) are displayed as the explicit water model. The initial structure of fluorophore was immersed in the center of a truncated octahedral box of TIP3P (Jorgensen et al., 1983) water molecules, and all of the PEG atoms were no less than 8 Å from the boundary of the water box. The relaxed structure was then gently heated from 0 to 300 K in 50 ps and equilibrated for 50 ps with weak restraints on each molecule, which was equilibrated for another 500 ps at constant pressure without restraint. Production simulations were extended to 140 ns for each molecule and trajectories were saved every 2 ps.

1.4 Centrifugal Filtration of Fluorophores

Considering the large aggregation of as-prepared fluorophores can be removed using centrifugal filter, fluorophores through filtration of 30, 50 and 100 kDa molecular weight cut-off (MWCO) are intravenously injected to investigate the effect of fluorophores aggregation on excretion behavior. The pass percentages of fluorophores through different filters are estimated with the optical density (OD) values at the peak and summarized in **Supplementary Table 3**. Selected time points from video-rate NIR-II imaging of mice in the supine positon after tail vein injection of as-prepared, 30 KDa and 100

KDa filtered **BGM6P** are measured (**Supplementary Figure S4-6**). It is found that fluorescence signal of liver can be detected for as-prepared **BGM6P** treated mouse at ~900 s post-injection (p. i.). By contrast, the florescence signal of liver is undetectable at ~200 s p.i. for mice with treatment of **BGM6P** after both 30 and 100 kDa filtration. It can also be observed that mice treated with 30 and 100 kDa filtered **BGM6P** exhibit urine signal peak at 400 s p. i., two time faster of excretion than 800 s for mouse treated with as-prepared **BGM6P**, and liver fluorescence intensity signal is counted with *ca.* 1000, 2500, 3500 at the time of urine signal peak for mice treated with 30, 100 kDa filtrated and as-prepared **BGM6P**, respectively (**Supplementary Figure S7**). These results demonstrate that fluorophores after centrifugal filtration display superior renal excretion ability and less liver accumulation than as-prepared fluorophores, suggesting fluorophores without large aggregation are more favorable for renal clearance pathway.

2 Synthetic Procedures and Characterization Data for the Molecular Fluorophores

The synthesis of **BGM6**, **BGP6** and **BGO6** and their PEGylated compounds **BGM6P**, **BGP6P** and **BGO6P** was shown in **Supplementary Scheme 1**, and the PEGylated fluorophores were characterized by size exclusion chromatography (SEC) (**Supplementary Figure 1**, **Supplementary Table 2**). PEG1500 (weight average molecular weight, M_w = 1500 g/mol) was conjugated to afford fluorophores water solubility.

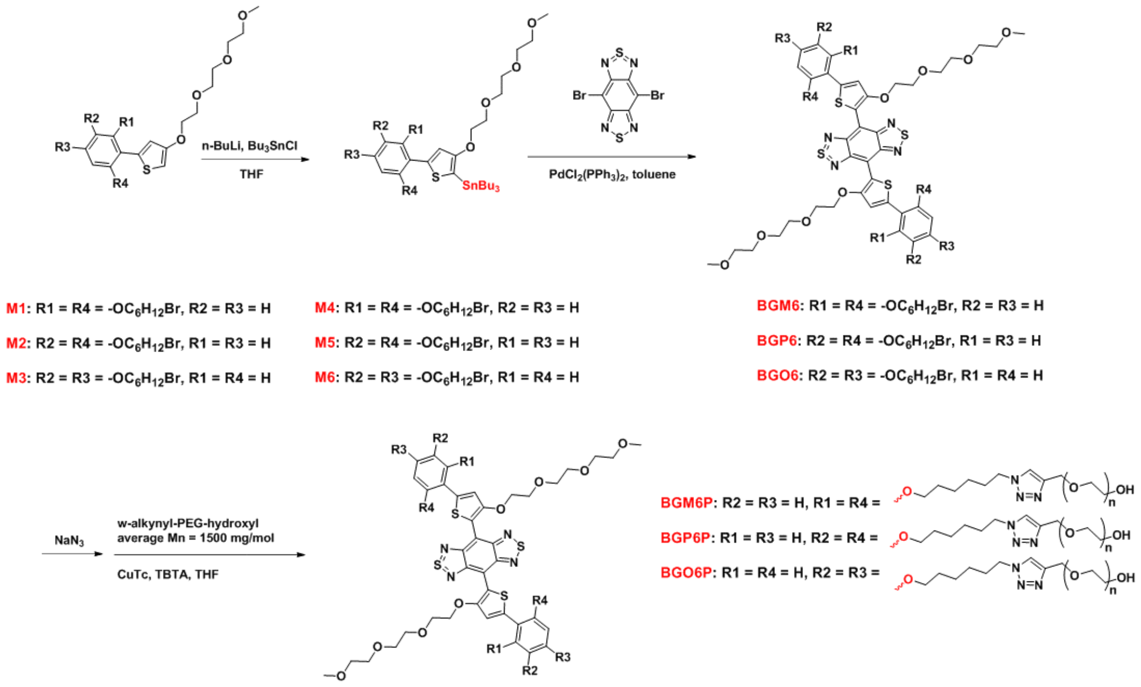

Supplementary Scheme 1. Synthetic routes of **BGM6**, **BGP6** and **BGO6** and their PEGylated compounds **BGM6P**, **BGP6P** and **BGO6P**.

The synthetic procedures of **M1-M3** were similar to the reported procedures in our previous work (Wan et al., 2018).

Compound **M1** (yield 53%). ^{1}H NMR (500 MHz, $CDCl_3$), δ (ppm) 7.19 (t, J = 6.5 Hz, 1H), 7.15 (d, J = 1.5 Hz, 1H), 6.58 (d, J = 1.5 Hz, 2H), 6.30 (d, J = 1.5 Hz, 1H), 4.16 (m, 2H), 3.99 (m, 4H), 3.88 (m, 2H), 3.75 (m, 2H), 3.76 (m, 2H), 3.70 (m, 2H), 3.68 (m, 2H), 3.57 (m, 4H), 3.40 (m, 3H), 1.89 (m, 4H), 1.83 (m, 4H), 1.55 (m, 8H). ^{13}C NMR (126 MHz, $CDCl_3$), δ (ppm) 157.35, 156.11, 133.25, 128.65, 121.22, 113.07, 105.46, 97.85, 72.08, 70.19, 70.81, 70.72, 69.94, 69.30, 68.98, 59.18, 33.99, 32.80, 29.07, 27.94, 25.46. HRMS (ESI) calcd for $C_{29}H_{45}O_6Br^{81}BrS$, ([M+$H_+$]) 681.1278, Found 681.1273.

Compound **M2** (yield 51%). ^{1}H NMR (500 MHz, $CDCl_3$), δ (ppm) 7.19 (s, 1H), 7.14 (d, J = 1.5 Hz, 1H), 6.87 (d, J = 1.5 Hz, 1H), 6.77 (s, 1H),6.27 (d, J = 1.5 Hz, 1H), 4.15 (m, 2H), 3.99 (m, 4H), 3.95 (m, 2H), 3.92 (m, 2H), 3.87 (m, 2H), 3.76 (m, 2H), 3.70 (m, 2H), 3.68 (m, 2H), 3.57 (m, 2H), 3.45 (m, 3H), 3.40 (m, 2H), 1.91 (m, 6H), 1.80 (m, 2H), 1.55 (m, 8H). ^{13}C NMR (126 MHz, $CDCl_3$), δ (ppm) 156.81, 153.15, 149.72, 138.12, 123.98, 117.12, 114.47, 114.11, 114.08, 98.32, 72.07, 70.93, 70.80, 70.72, 69.86, 69.47, 69.40, 68.53, 59.20, 34.01, 33.99, 32.83, 32.78, 31.09, 29.32, 28.08, 28.02, 25.56, 25.44. HRMS (ESI) calcd for $C_{29}H_{45}O_6Br^{81}BrS$, ([M+$H_+$]) 681.1278, Found 681.1273.

Compound **M3** (yield 64%). ^{1}H NMR (500 MHz, $CDCl_3$), δ (ppm) 7.04 (d, J = 1.5 Hz, 1H), 6.89 (d, J = 1.5 Hz, 1H), 6.85 (d, J = 1.5 Hz, 1H), 6.83 (s, 1H),6.15 (d, J = 1.5 Hz, 1H), 4.14 (m, 2H), 4.04 (m, 2H), 3.98 (m, 2H), 3.85 (m, 2H), 3.83 (m, 2H), 3.74 (m, 4H), 3.70 (m, 2H), 3.66 (m, 2H), 3.64 (m, 4H), 3.40 (m, 2H), 1.91-1.81 (m, 8H), 1.54 (m, 8H). ^{13}C NMR (126 MHz, $CDCl_3$), δ (ppm) 157.45, 149.26, 149.14, 142.93, 127.80, 118.43, 115.02, 114.07, 111.61, 96.35, 72.04, 70.91, 70.77, 70.68, 69.79, 69.44, 69.20, 69.16, 59.14, 33.87, 32.84, 32.82, 29.22, 29.20, 28.04, 28.02, 25.38, 25.37. HRMS (ESI) calcd for $C_{29}H_{45}O_6Br^{81}BrS$, ([M+$H_+$]) 681.1278, Found 681.1271.

The intermediates **M4-M6** were prepared with the similar procedures as follow.

General procedures for **M4-M6**. To a solution of compound **M1-M3** (680 mg, 1.0 mmol) in 60 mL THF at -78 ^{o}C under protection gas atmosphere, *n*-BuLi (1.6 M in hexane, 0.69 mL, 1.1 mmol) was added dropwise. After stirring at this temperature for another 1.5 h, tributyltin chloride (340 mg, 1.2

mmol) was added to the solution. Then the reaction was slowly warmed to room temperature and stirred overnight. After that, the mixture was poured into water and extracted twice with ethyl acetate, the combined organic phase was dried with $MgSO_4$ and evaporated in vacuo without further purification.

Product **BGM6**, **BGP6** and **BGO6** were prepared with the similar procedures.

General procedures for **BGM6**, **BGP6** and **BGO6**. To a solution of the crude compound **M4-M6** (1.1g, about 1.12 mmol) and BBT-Br (157 mg, 0.45 mmol) in toluene (15 mL) under protection gas atmosphere, $Pd(PPh_3)_2Cl^2$ (140 mg) was added. The mixture was stirred at 120 ºC for 12 h. After cooling to room temperature, the mixture was poured into water and extracted twice with ethyl acetate. The organic phase was dried with $MgSO_4$ and evaporated in vacuo. The crude material was purified by silica gel column chromatography (PE/DCM = 4:1) to afford compound **BGM6**, **BGP6** and **BGO6** as dark green solid (yield ~45%).

Compound **BGM6** (yield 43%). ^{1}H NMR (500 MHz, $CDCl_3$), δ (ppm) 7.80 (s, 2H), 7.26-7.18 (m, 2H), 6.66 (d, J = 1.5 Hz, 2H), 4.36-4.33 (t, J = 6.5 Hz, 4H), 4.10-4.07 (m, 8H), 3.71-3.68 (m, 4H), 3.57-3.53 (m, 12H), 3.49-3.47 (m, 4H), 3.36-3.32 (m, 14H), 1.93-1.80 (m, 16H), 1.57-1.49 (m, 16H). ^{13}C NMR (126 MHz, $CDCl_3$), δ (ppm) 157.29, 154.87, 153.08, 137.02, 128.69, 119.63, 114.75, 114.12, 112.83, 105.45, 72.00, 70.88, 70.79, 70.65, 70.61, 70.23, 69.19, 59.12, 33.96, 32.80, 29.22, 28.07, 25.66. HRMS (ESI) calcd for $C64H87O12N4Br^{81}BrS_4$, ([M+H+]) 1551.1890, Found 1551.1881.

Compound **BGP6** (yield 47%). ^{1}H NMR (500 MHz, $CDCl_3$), δ (ppm) 7.57 (s, 2H), 7.31 (s, 1H), 6.93-6.90 (d, J = 1.5 Hz, 1H), 6.84-6.81 (m, 1H), 4.40-4.38 (t, J = 6.5 Hz, 2H), 4.10-4.07 (m, 2H), 4.01-3.99 (m, 2H), 3.71-3.69 (m, 2H), 3.58-3.53 (m, 6H), 3.49-3.46 (m, 4H), 3.37-3.32 (m, 5H), 1.94-1.82 (m, 8H), 1.57-1.49 (m, 8H). ^{13}C NMR (126 MHz, $CDCl_3$), δ (ppm) 155.73, 153.21, 152.98, 150.01,, 141.44, 123.81, 115.96, 115.21, 114.75, 114.04, 113.94, 72.05, 71.98, 71.05, 70.79, 70.65, 70.61, 70.16, 69.50, 68.61, 59.13, 34.02, 33.95, 32.83, 32.79, 29.38, 28.10, 26.83, 25.68, 25.48. HRMS (ESI) calcd for $C64H87O12N4Br^{81}BrS_4$, ([M+H+]) 1551.1890, Found 1551.1884.

Compound **BGO6** (yield 42%). ^{1}H NMR (500 MHz, $CDCl_3$), δ (ppm) 7.25 (d, J = 1.5 Hz, 1H), 7.20 (d, J = 1.7 Hz, 1H), 6.92-6.91 (d, J = 1.5 Hz, 1H), 4.41-4.39 (t, J = 6.5 Hz, 2H), 4.10-4.04 (m, 4H), 3.70-3.68 (m, 2H), 3.59-3.56 (m, 2H), 3.53 (m, 2H), 3.51-3.34 (m, 11H), 1.94-1.84 (m, 8H), 1.57 (m, 8H). ^{13}C NMR (126 MHz, $CDCl_3$), δ (ppm) 156.49, 152.82, 149.59, 149.27, 146.38, 127.53, 118.70, 113.82, 113.78, 113.46, 111.50, 71.96, 71.16, 70.78, 70.64, 70.60, 70.12, 69.28, 69.06, 59.12, 45.14,

33.94, 32.84, 32.82, 29.25, 29.20, 28.06, 28.04, 25.41, 25.39. HRMS (ESI) calcd for C64H87O12N4Br^{81}BrS_4, ([M+H+]) 1551.1890, Found 1551.1919.

Product **BGM6P**, **BGP6P** and **BGO6P** were prepared with the similar procedures as follow.

General procedures for **BGM6P**, **BGP6P** and **BGO6P**. **BGM6**, **BGP6** or **BGO6** (84 mg, 0.054 mmol) and sodium azide (50 mg, 0.75 mmol) were dissolved in DMF (10 mL), and the mixture was stirred for 3 h at room temperature. Then a large amount of water was added until all solids were dissolved. The reaction was extracted twice with ethyl acetate, the combined organic phase was dried with $MgSO_4$ and evaporated in vacuum. The crude product was subjected to flash column chromatography on silica gel to afford a dark green solid (69 mg, 0.052mmol). The dark green solid was dissolved in THF (5 mL) and copper (I) thiophene-2-carboxylate (CuTc) (5 mg), w-alkynyl-PEG-hydroxyl PEG1500 (*Mn* = 1500 mg/mL) (150 mg, about 0.1 mol) and tris[(1-benzyl-1H-1,2,3-triazol-4-yl)methyl]amine (TBTA) (3 mg) were added. The system was stirred at room temperature for 0.5 h, and then filtered with diatomite, and the solution was evaporated in vacuum. The crude product was purified by thin layer chromatography twice. First, ethyl acetate was used as an eluent and a small amount of impurities would move to the top of the TLC plate, but other parts of product remained at the start point of the TLC plate. Then DCM/MeOH (10:1-5:1) was used as an eluent successively, and the PEGylated product could be separated from alkyne-PEG (yiled ~85%).

BGM6P (yield 86%). SEC measured: *Mn* = 7191 g/mol, *Mw* = 7676 g/mol, PDI = 1.067.

BGP6P (yield 83%). SEC measured: *Mn* = 7523 g/mol, *Mw* = 8006 g/mol, PDI = 1.064.

BGO6P (yield 89%). SEC measured: *Mn* = 7486 g/mol, *Mw* = 7953 g/mol, PDI = 1.063.

3 Supplementary Figures and Tables

3.1 Supplementary Figures

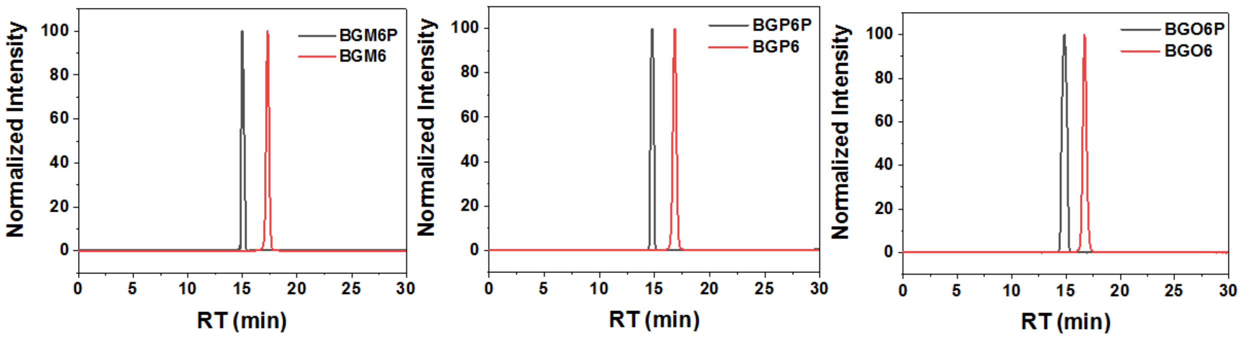

Supplementary Figure 1. Size-exclusion chromatography (SEC) analysis of the un-PEGylated and PEGylated molecular fluorophores.

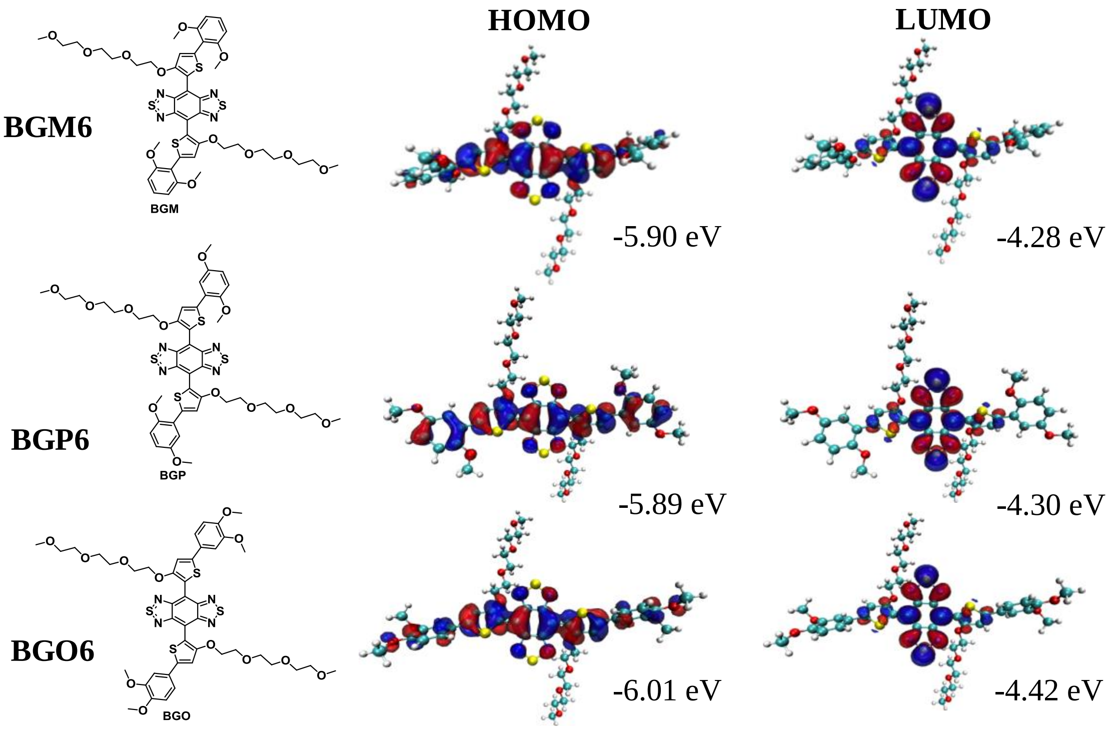

Supplementary Figure 2. Calculated HOMOs and LUMOs of the molecular fluorophores at the tuned-ωB97XD*/6-31G(d) level. The HOMO and LUMO energy levels are also presented in the figures. To reduce the computational requirements, side chains on the benzene units are replaced by methyloxy groups. Note that the LUMO levels are obtained by subtracting the optical gap from the HOMO levels.

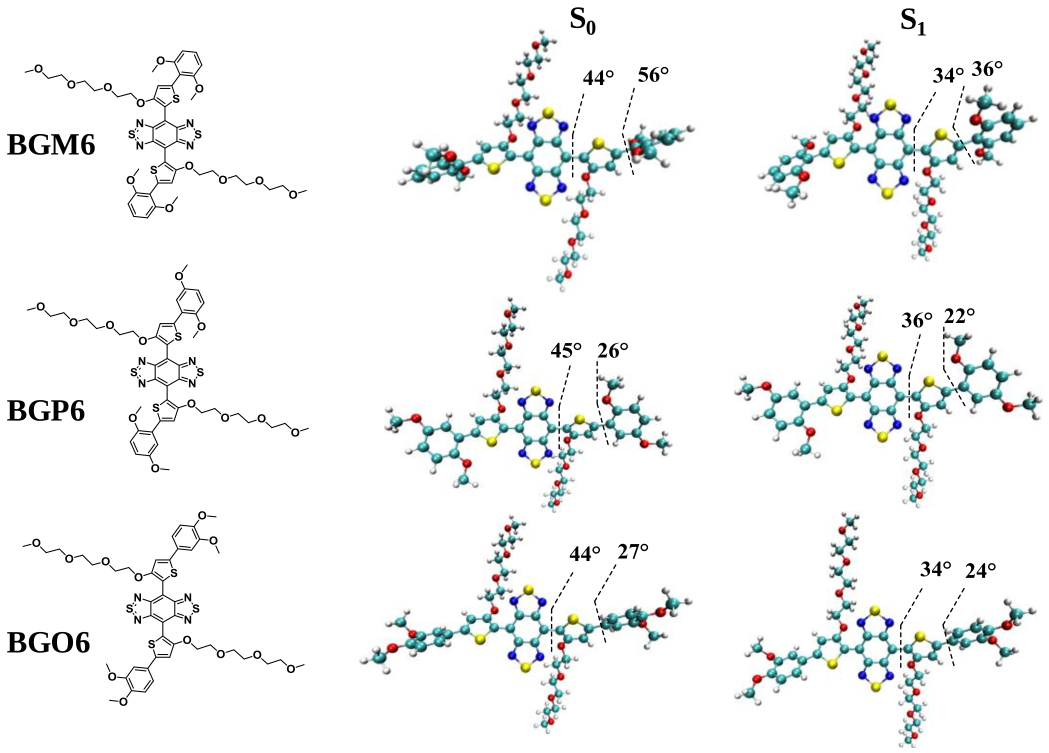

Supplementary Figure 3. Optimized ground-state (S_0) and first singlet excited state (S_1) geometries of the molecular fluorophores at the optimally tuned ωB97XD*/6-31G(d) level. To reduce the computational requirements, side chains on the fluorene units are replaced by methyloxy groups. The dihedral angles are shown.

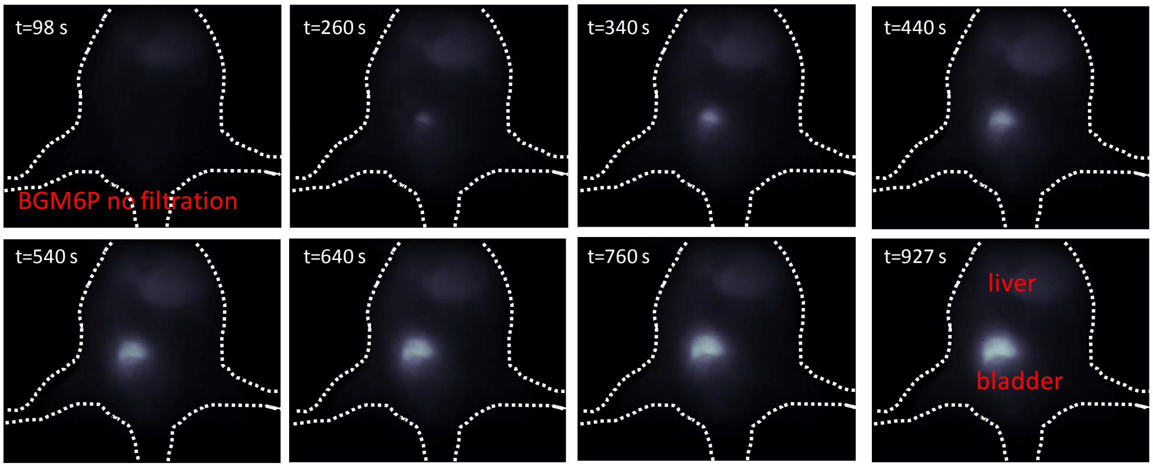

Supplementary Figure 4. Selected time points from video-rate NIR-II imaging of a mouse in the supine positon after tail vein injection of as-prepared **BGM6P** (Inject dose: OD=2, 200 uL, 50 ms exposure time).

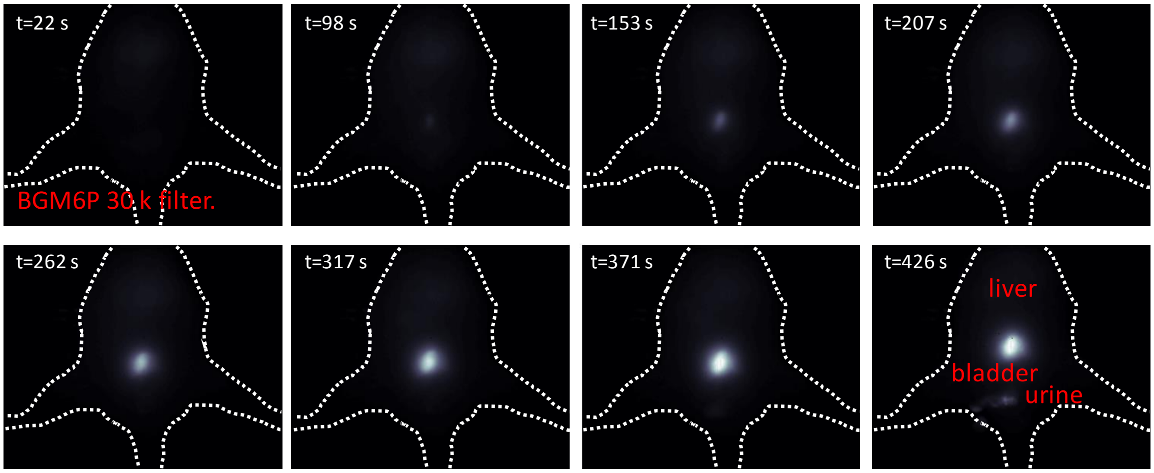

Supplementary Figure 5. Selected time points from video-rate NIR-II imaging of a mouse in the supine positon after tail vein injection of 30 K filtered **BGM6P**.

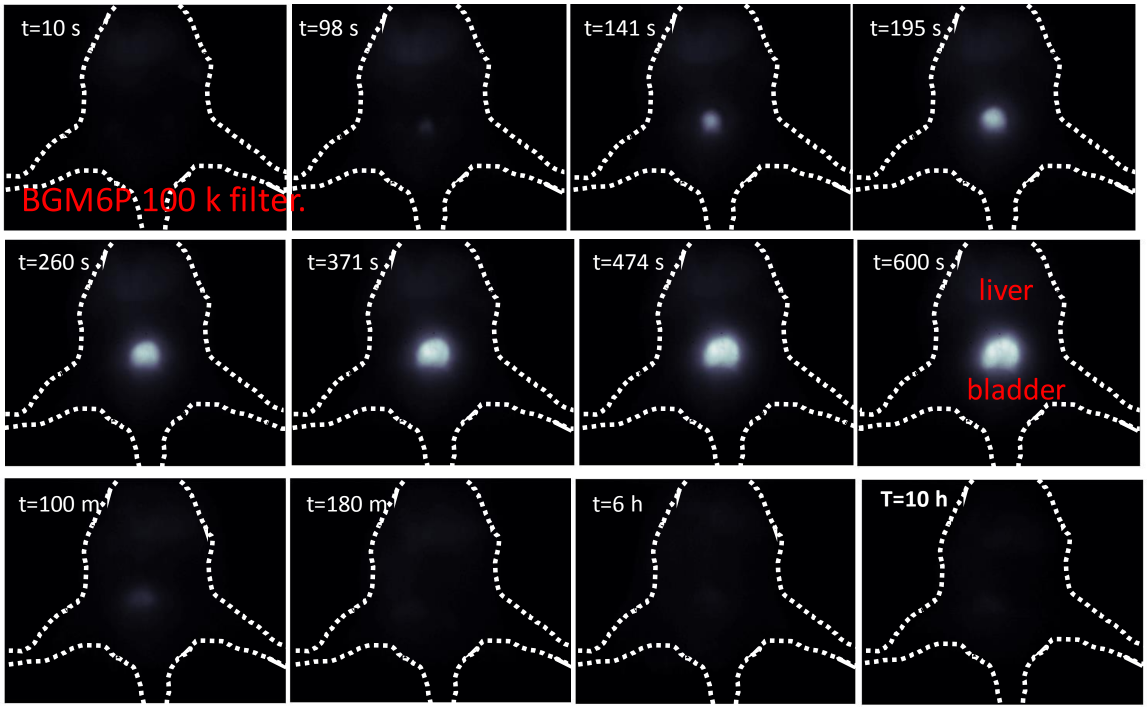

Supplementary Figure 6. Selected time points from video-rate NIR-II imaging of a mouse in the supine positon after tail vein injection of 100 K filtered **BGM6P**.

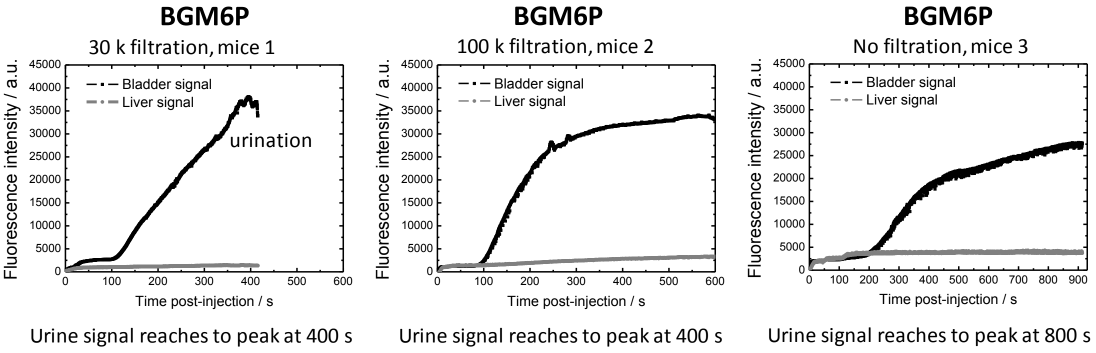

Supplementary Figure 7. Bladder and liver signal of the mouse with an injection of 30 K filtered, 100 K filtered and as-prepared **BGM6P**. Liver signal is *ca.* 1000, 2500, 3500 counts, respectively.

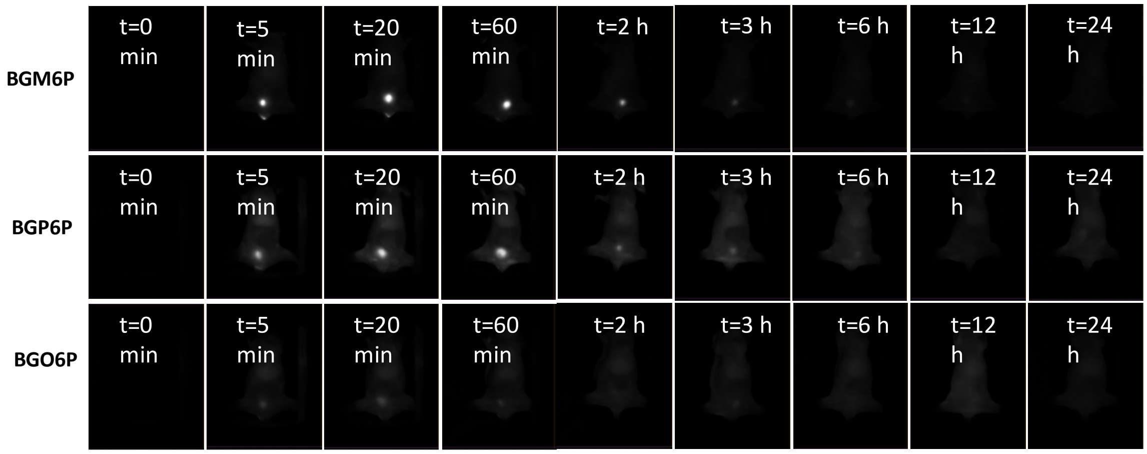

Supplementary Figure 8. Selected time points from NIR-II whole body imaging of mice in the supine positon after tail vein injection of 30 K filtrated **BGM6P**, **BGP6P** and **BGO6P**, respectively.

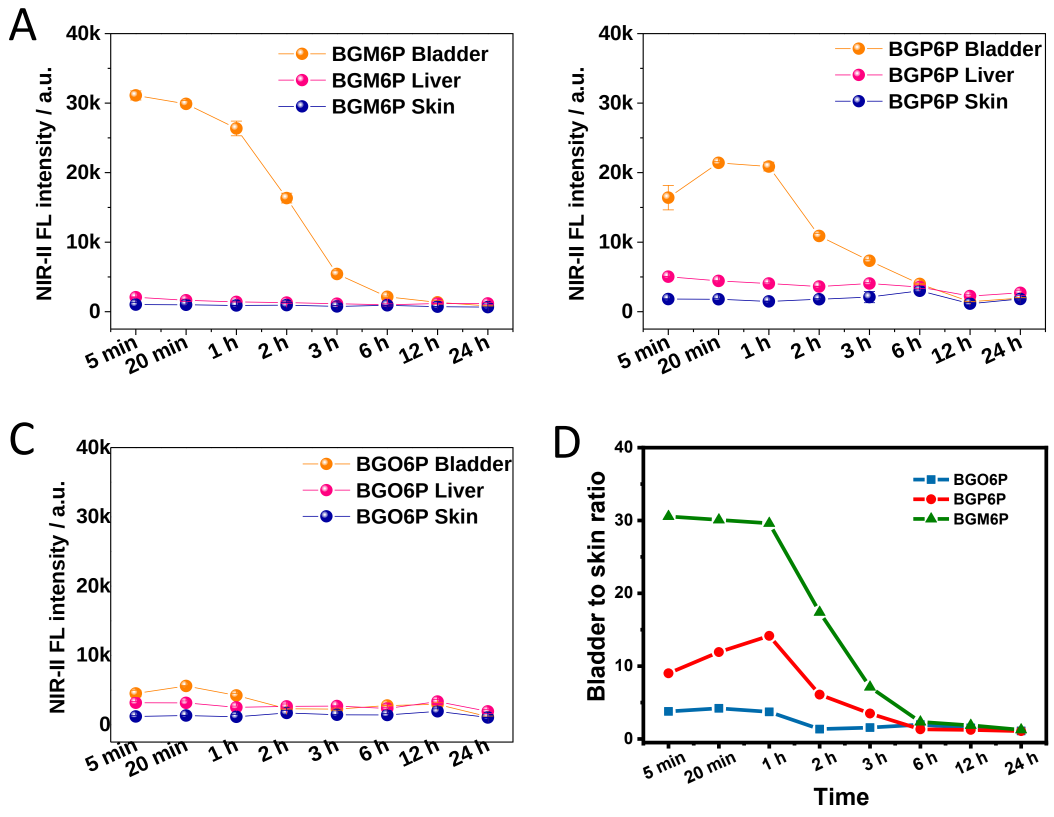

Supplementary Figure 9. NIR-II fluorescent signal intensity of liver (A), bladder (B) and skin (C) regions for **BGO6P**, **BGP6P** and **BGM6P** injected mouse at different time after injection. (D) Representative background subtracted signal of bladder to skin as a function of time for mice injected with **BGM6P**, **BGP6P** and **BGO6P**, respectively.

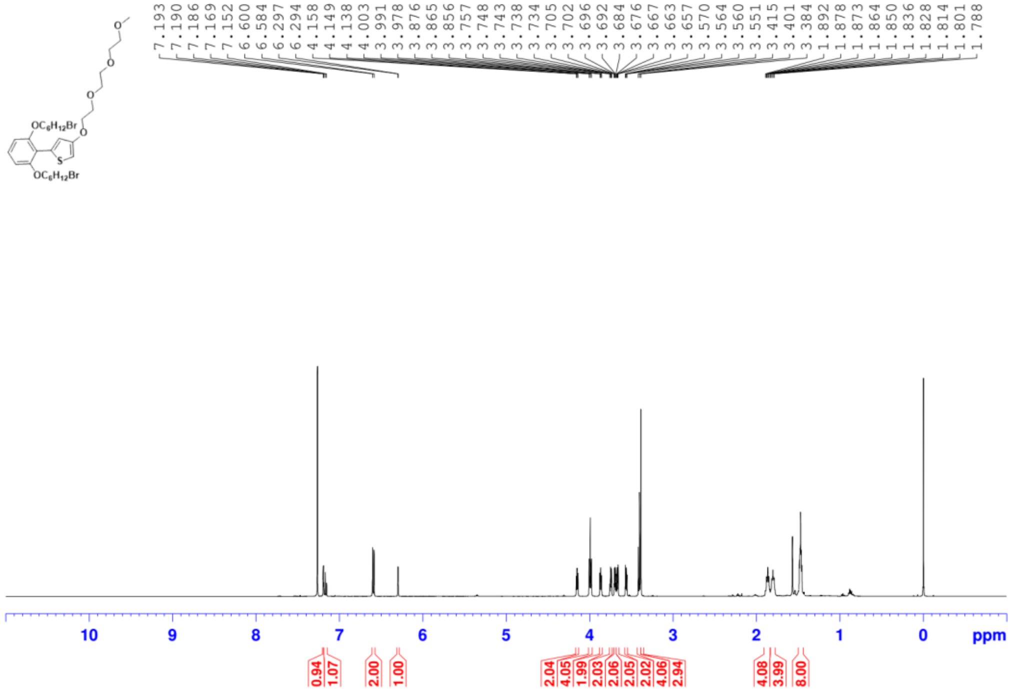

Supplementary Figure 10. ^{1}H NMR spectrum of **M1**.

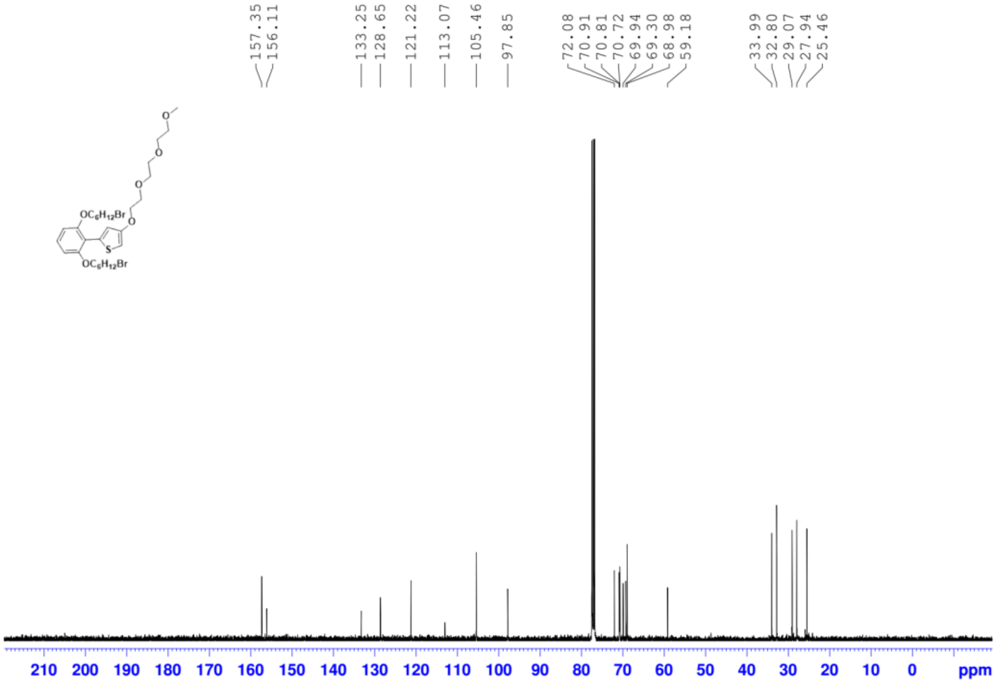

Supplementary Figure 11. ^{13}C NMR spectrum of **M1**.

Positive:

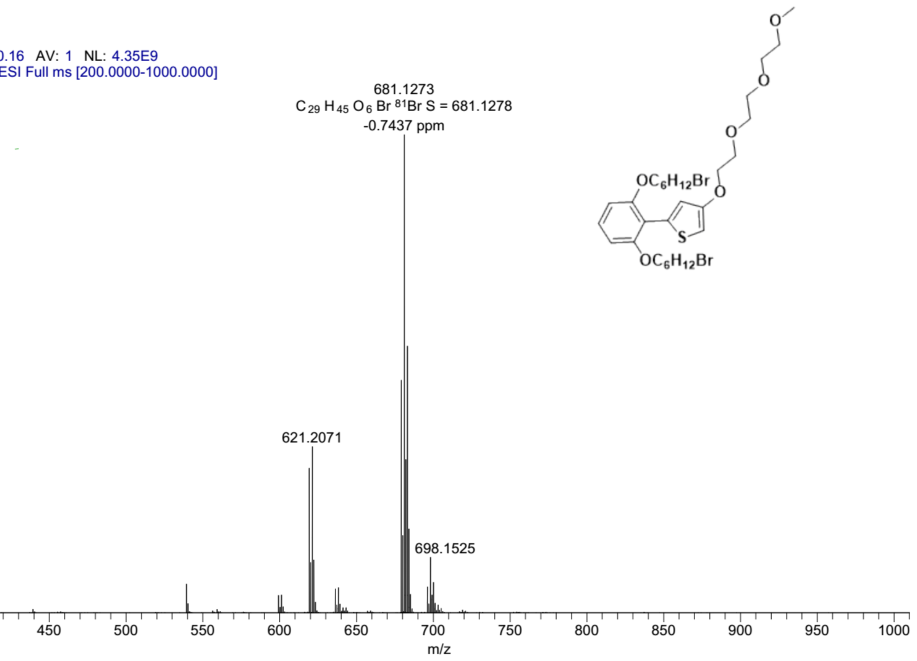

[M+H]

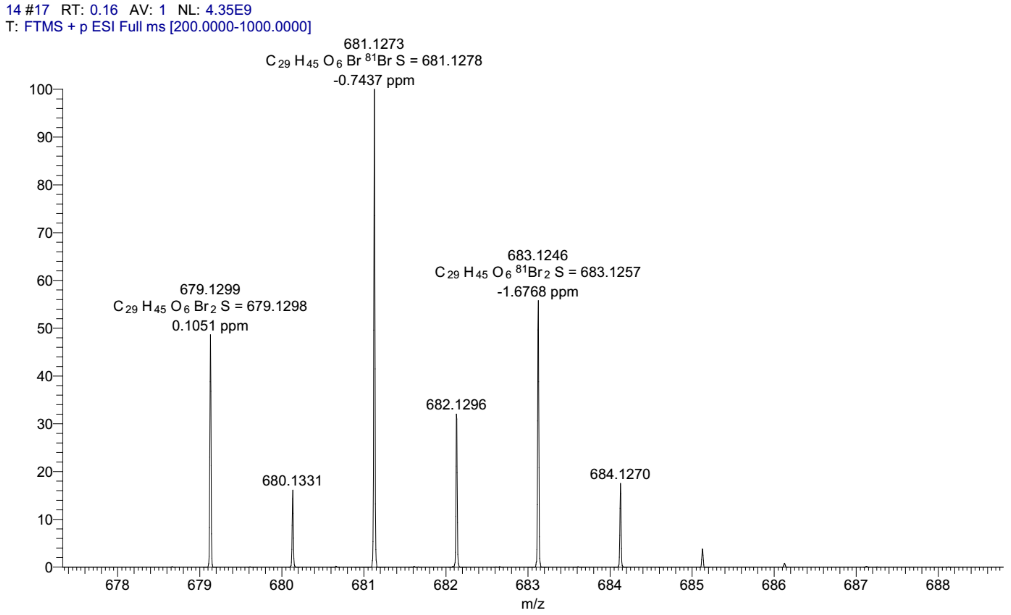

Supplementary Figure 12. HR MASS spectrum of **M1**.

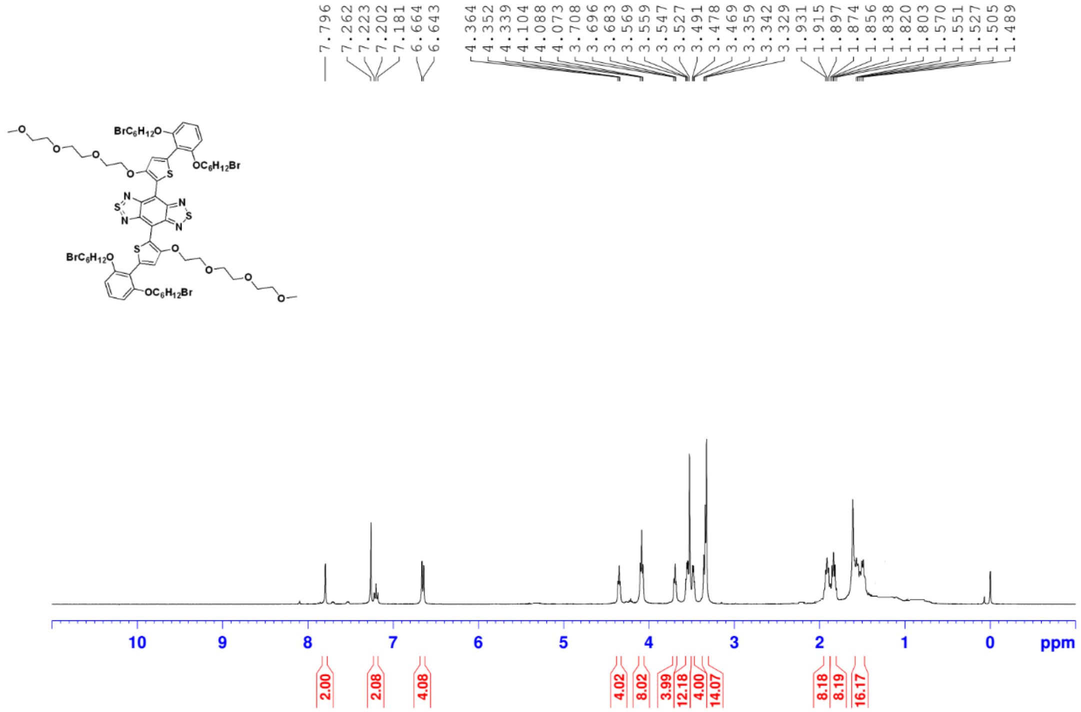

Supplementary Figure 13. ^{1}H NMR spectrum of **BGM6**.

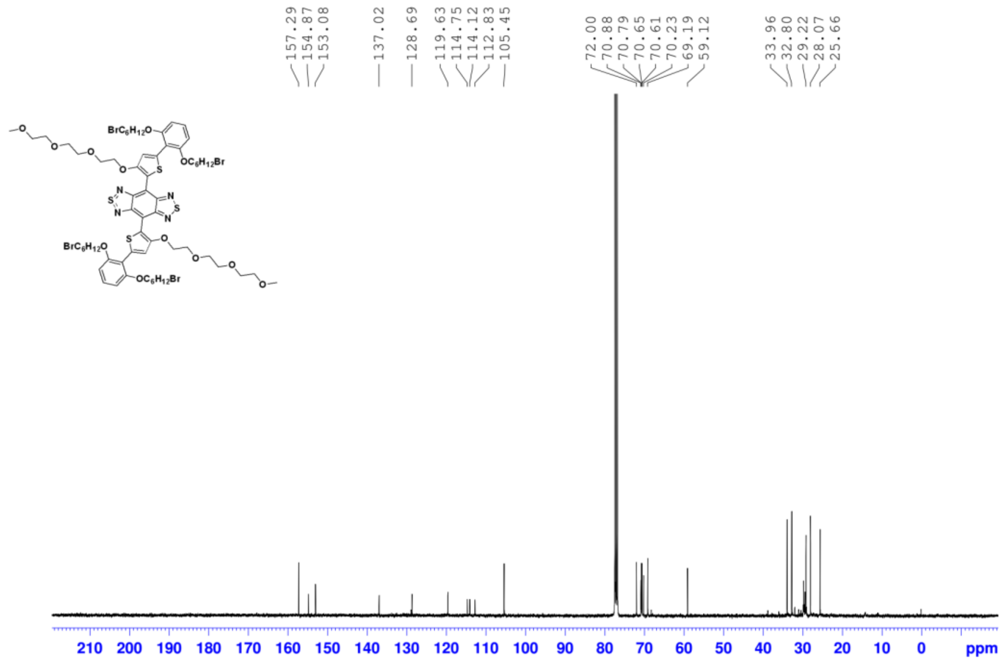

Supplementary Figure 14. ^{13}C NMR spectrum of **BGM6**.

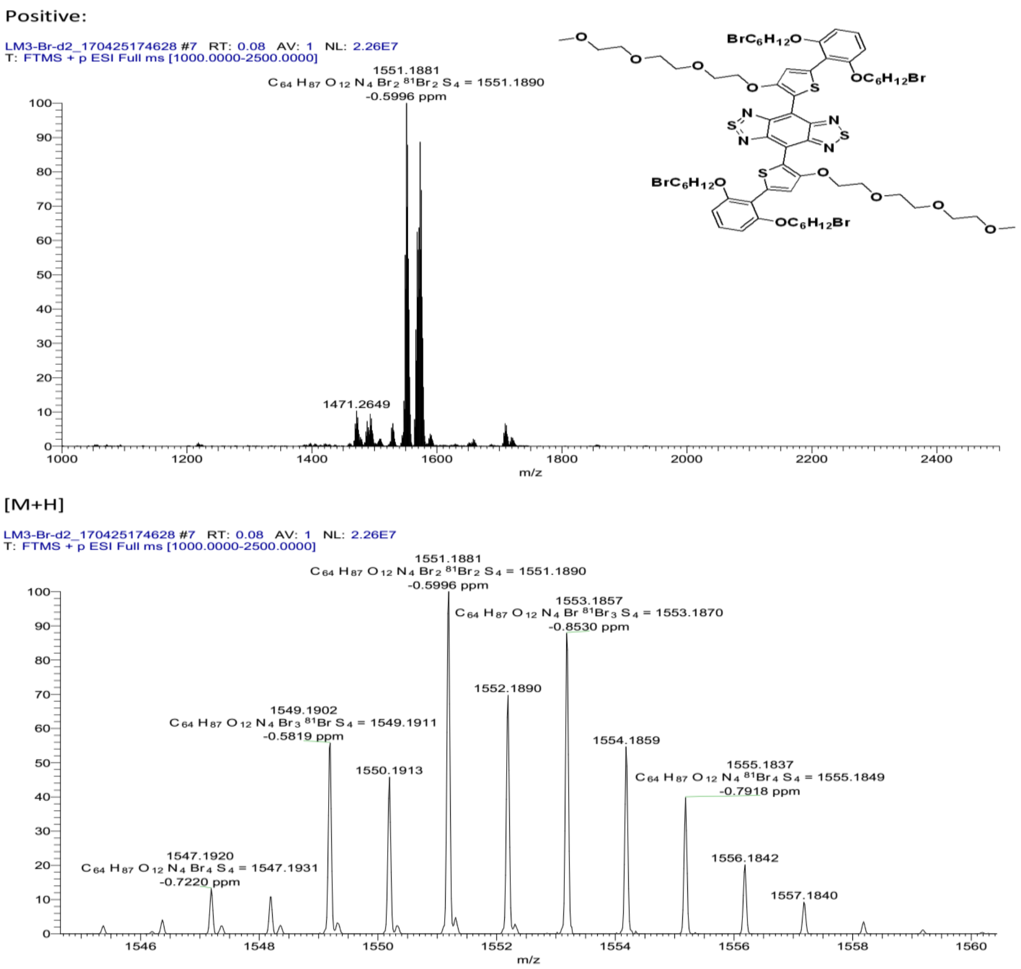

Supplementary Figure 15. HR MASS spectrum of **BGM6**.

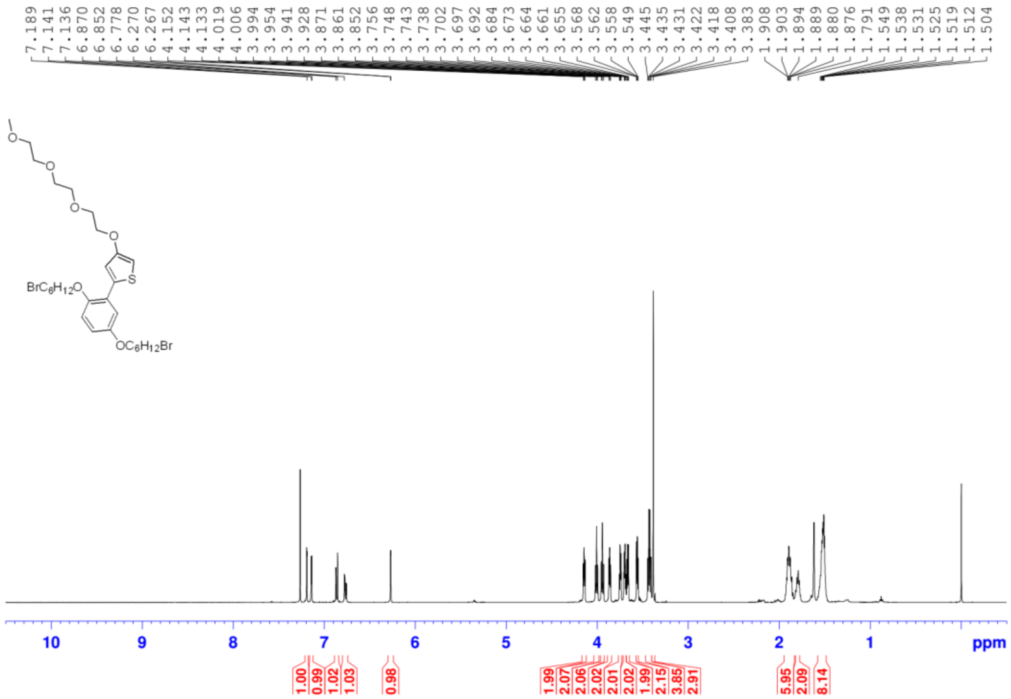

Supplementary Figure 16. ^{1}H NMR spectrum of **M2**.

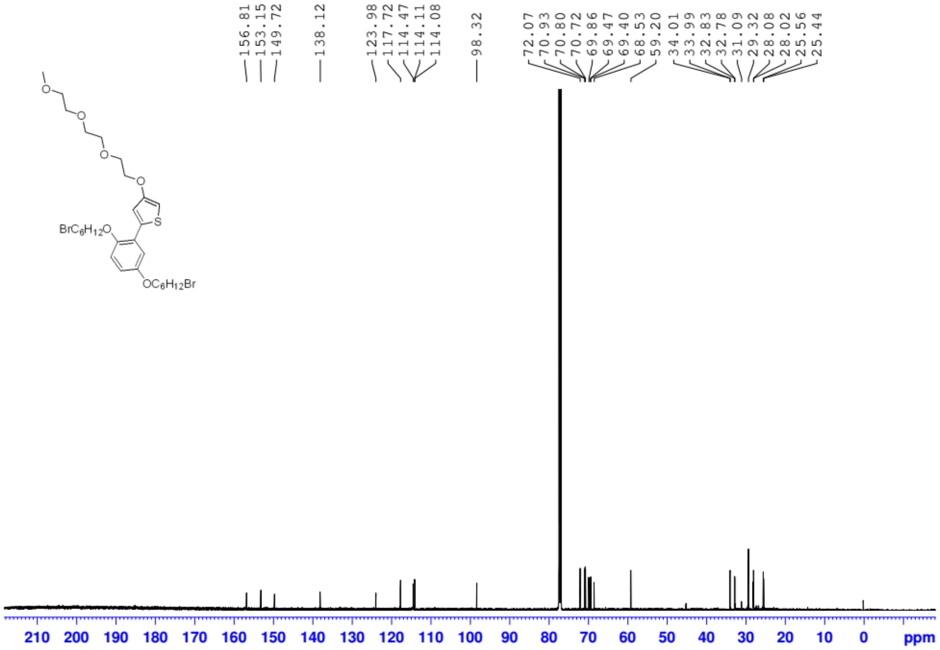

Supplementary Figure 17. ^{13}C NMR spectrum of **M2**.

Positive:

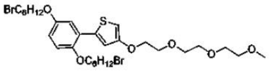

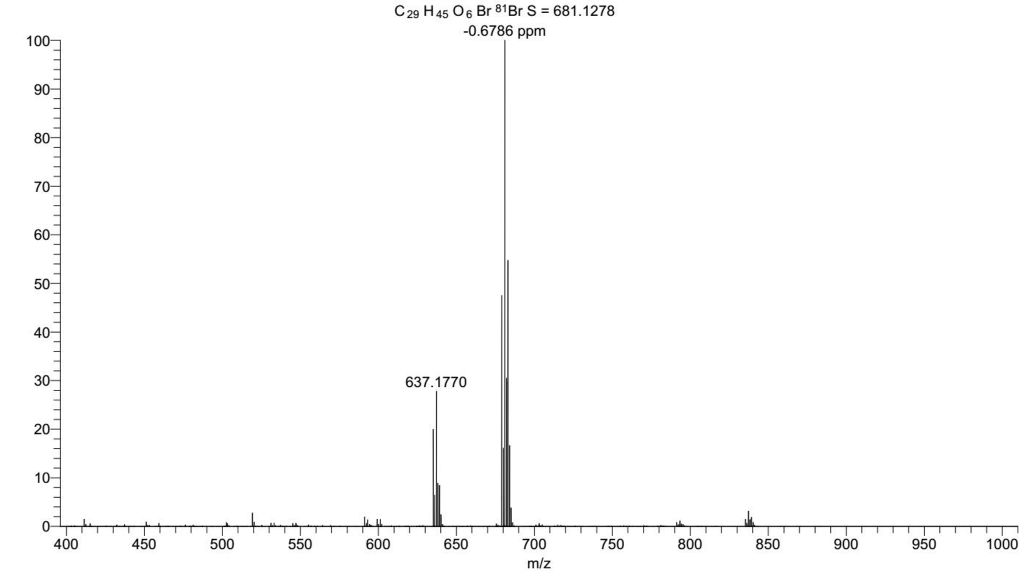

[M+H]

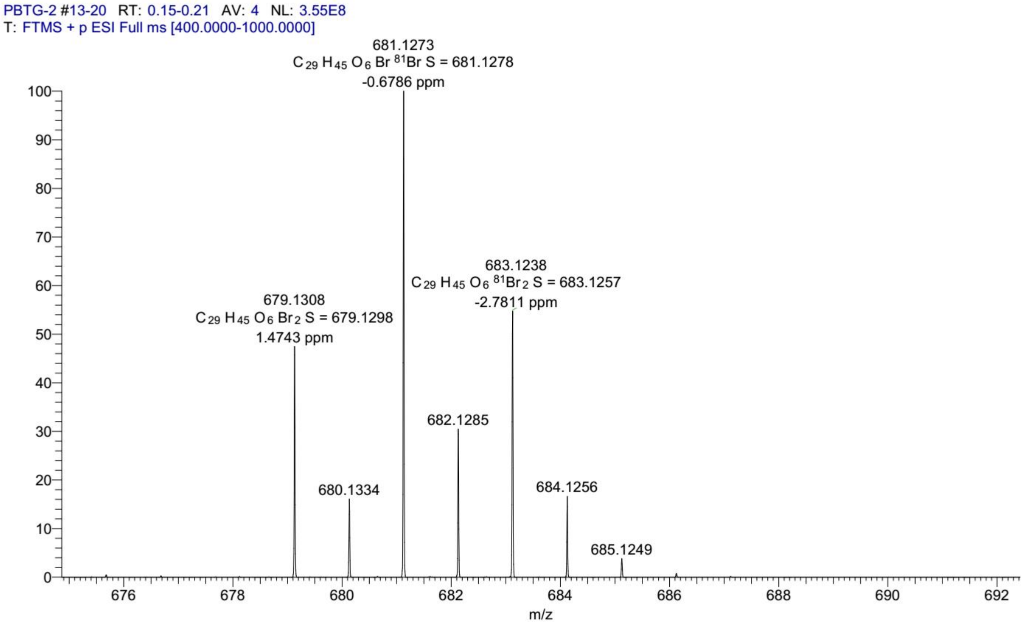

Supplementary Figure 18. HR MASS spectrum of **M2**.

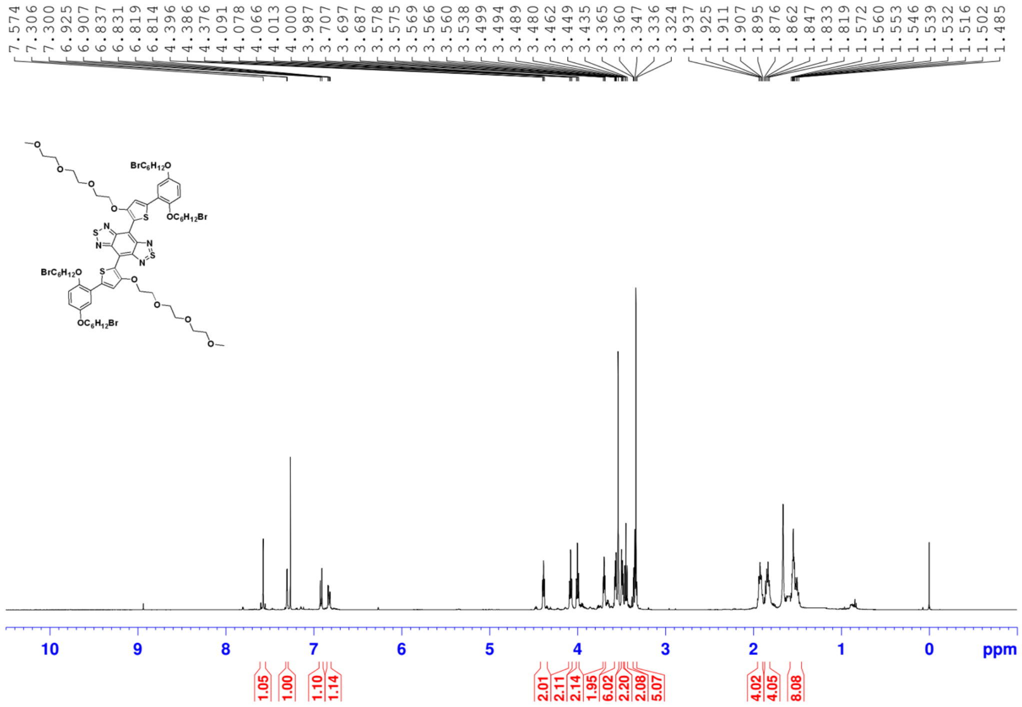

Supplementary Figure 19. ^{1}H NMR spectrum of **BGP6**.

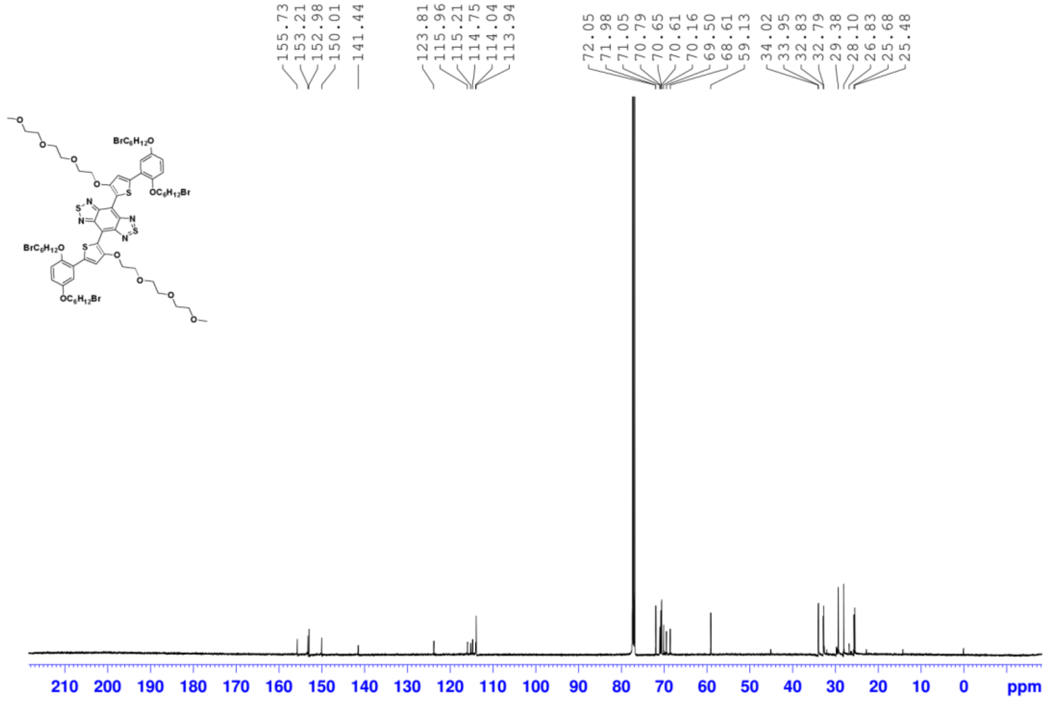

Supplementary Figure 20. ^{13}C NMR spectrum of **BGP6**.

Positive:

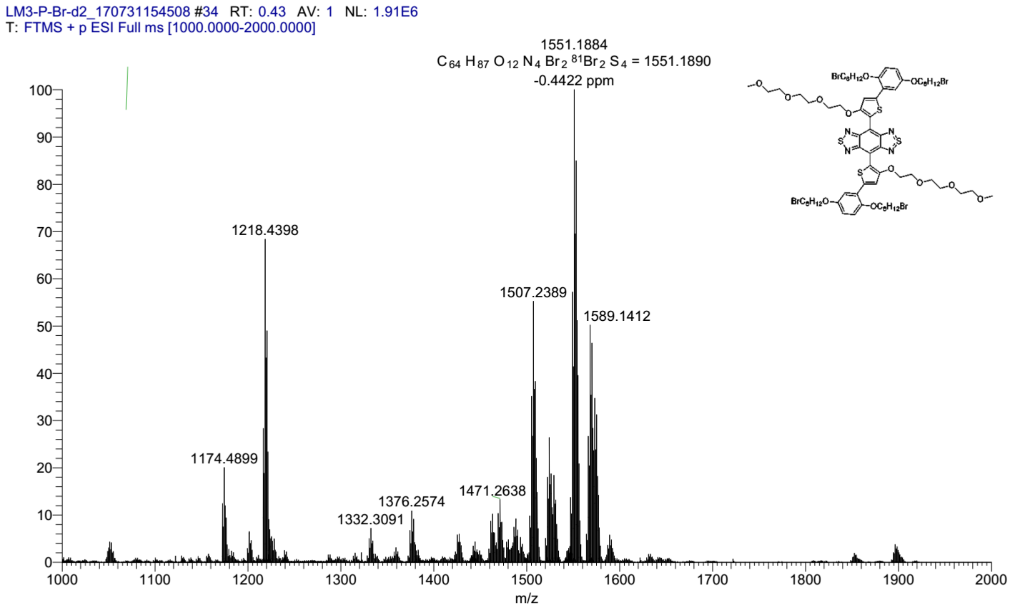

[M+H]

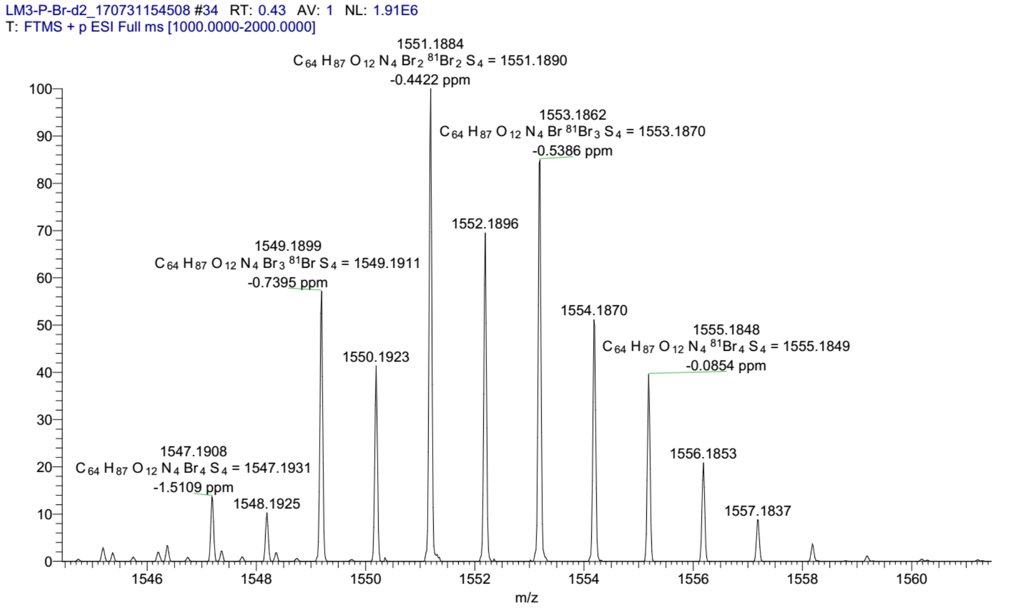

Supplementary Figure 21. HR MASS spectrum of **BGP6**.

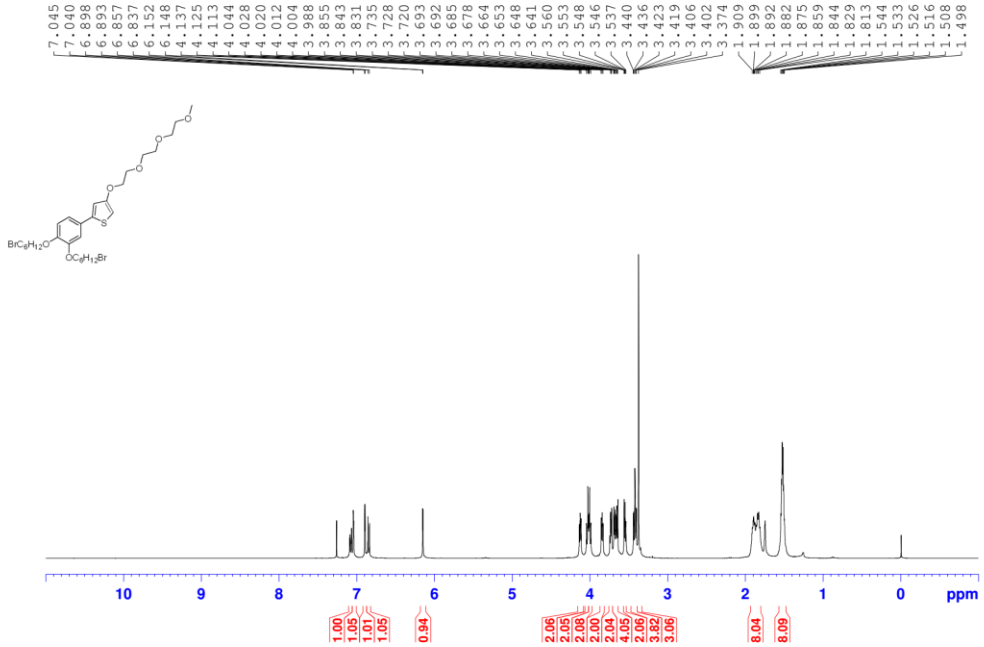

Supplementary Figure 22. ^{1}H NMR spectrum of **M3**.

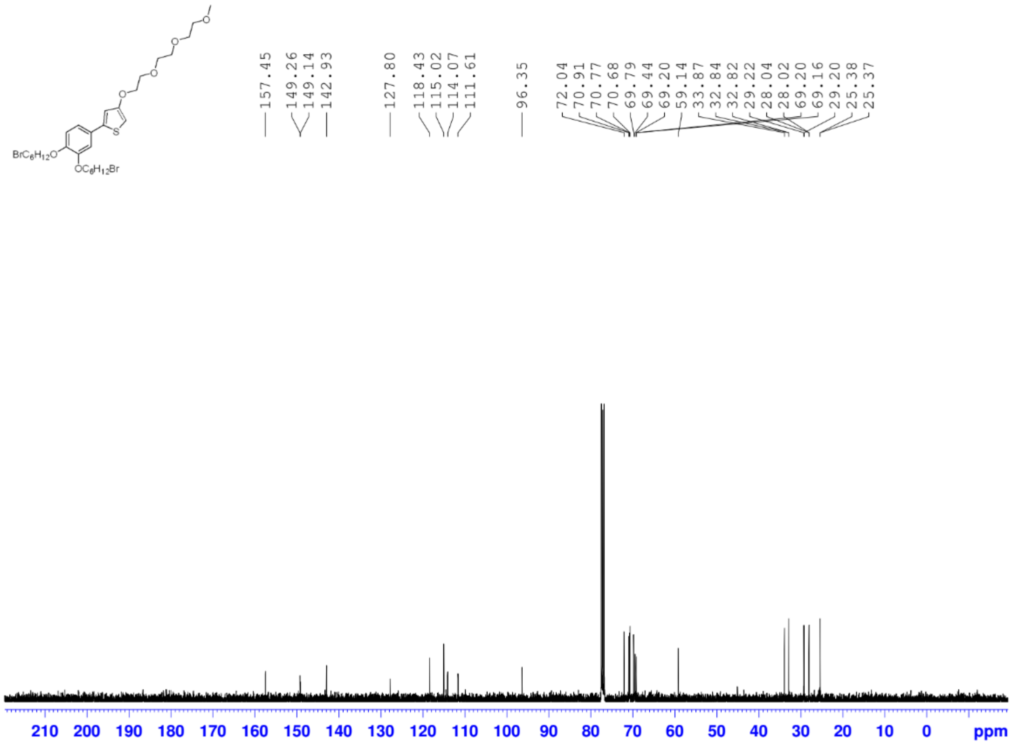

Supplementary Figure 23. ^{13}C NMR spectrum of **M3**.

Positive:

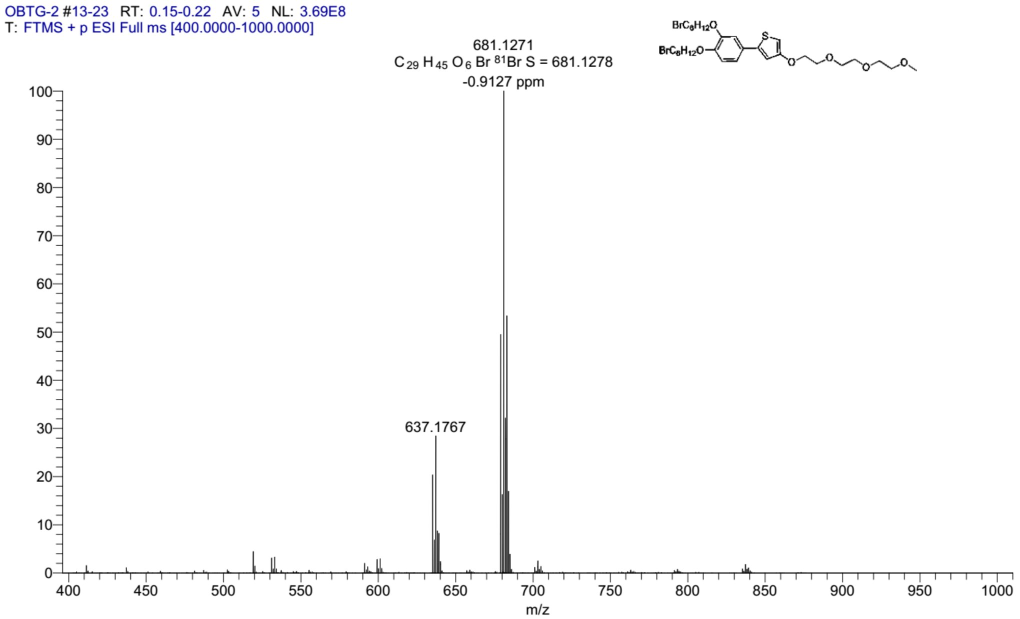

[M+H]

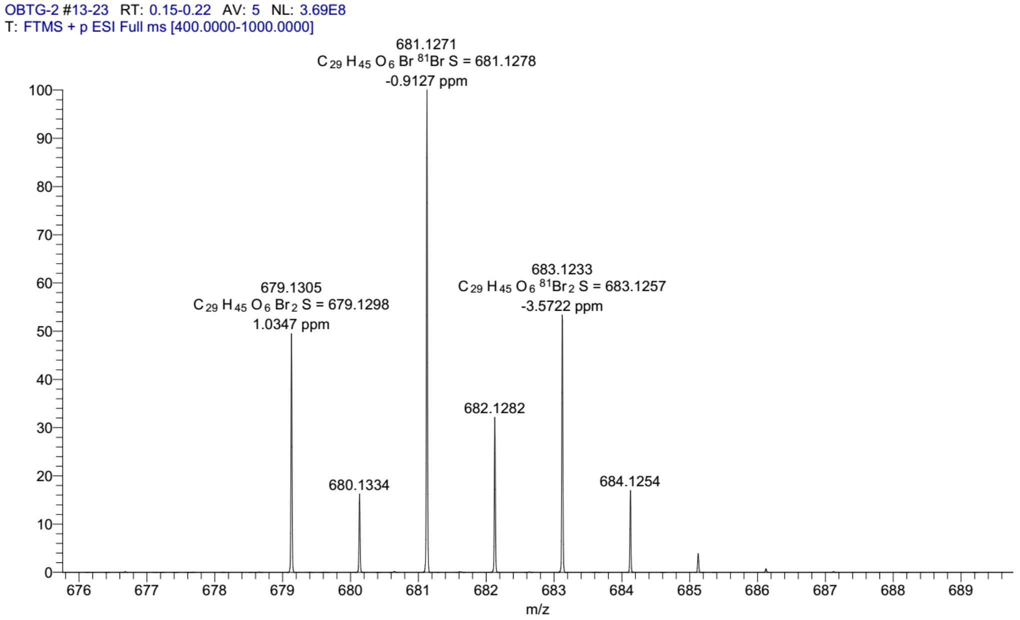

Supplementary Figure 24. HR MASS spectrum of **M3**.

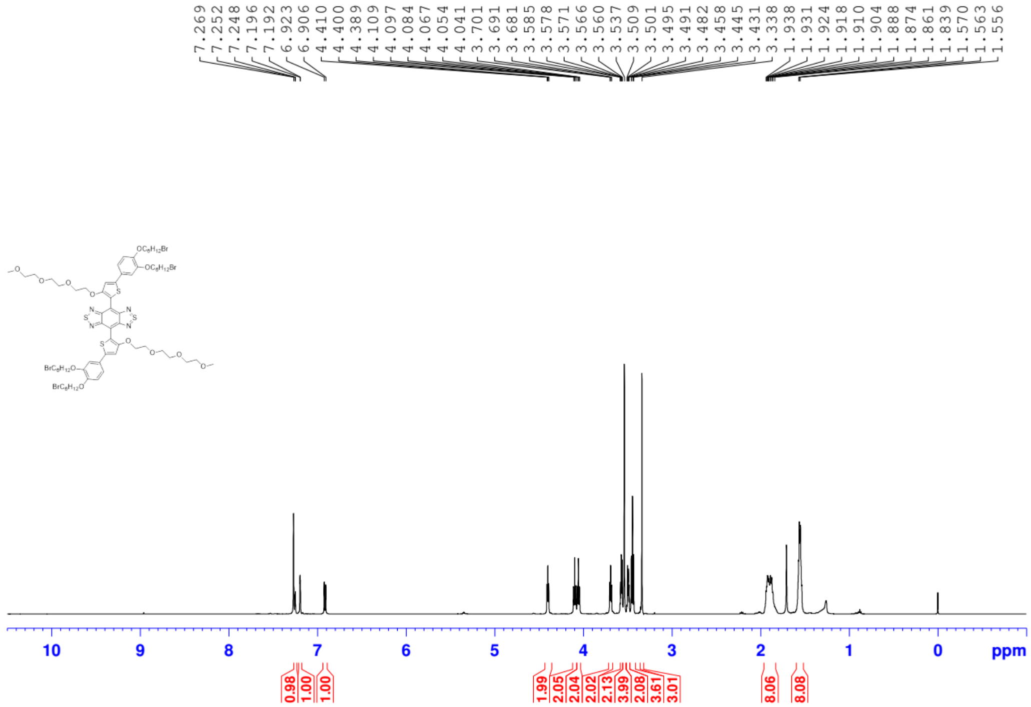

Supplementary Figure 25. ^{1}H NMR spectrum of **BGO6**.

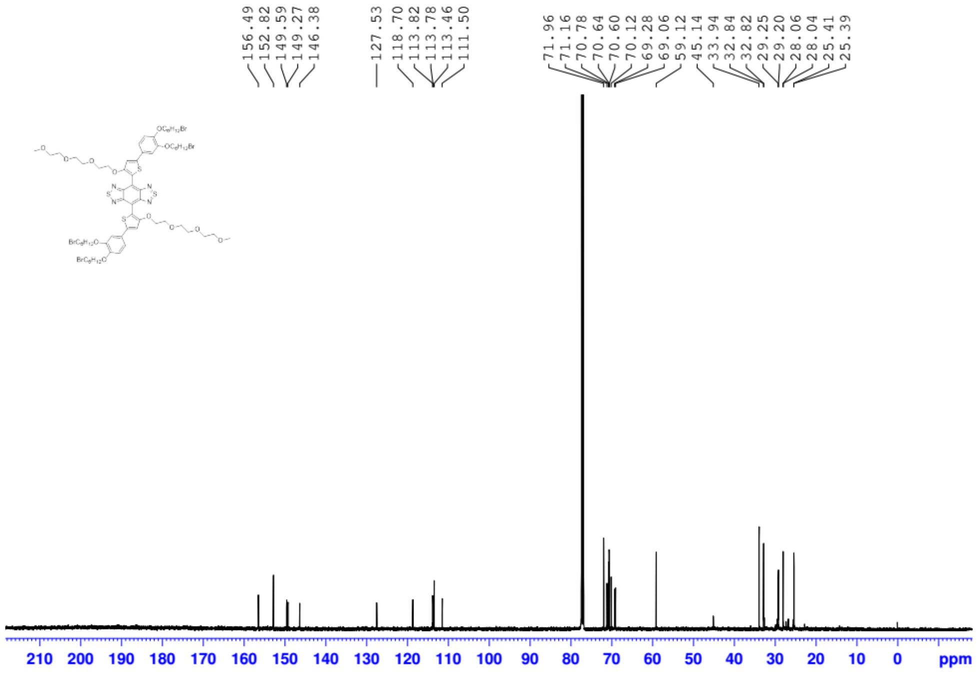

Supplementary Figure 26. ^{13}C NMR spectrum of **BGO6**.

Positive:

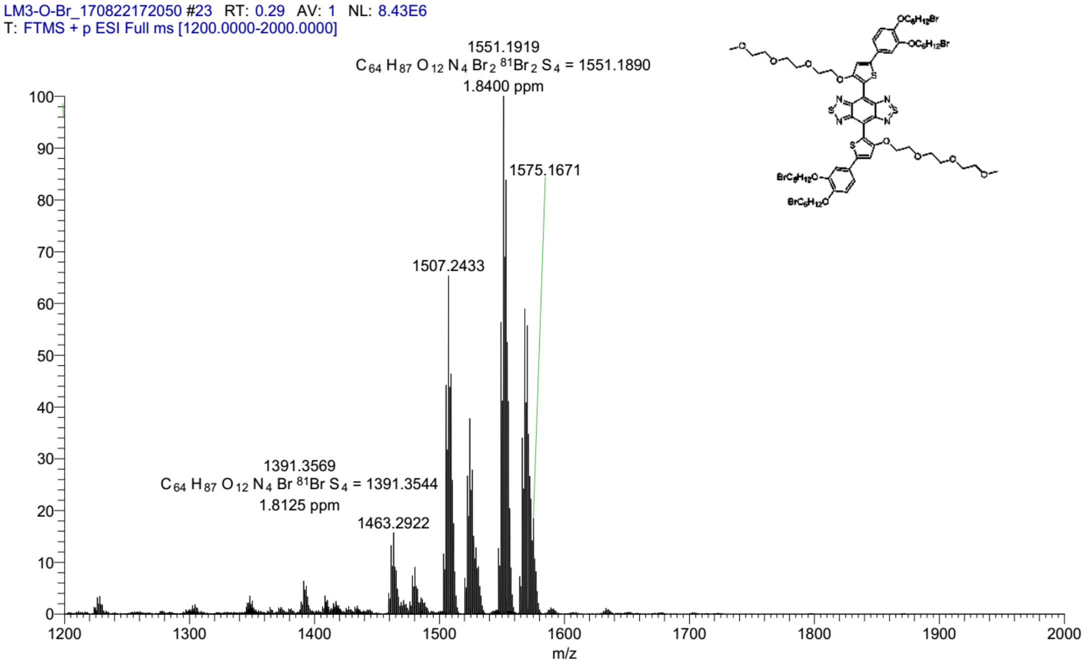

[M+H]

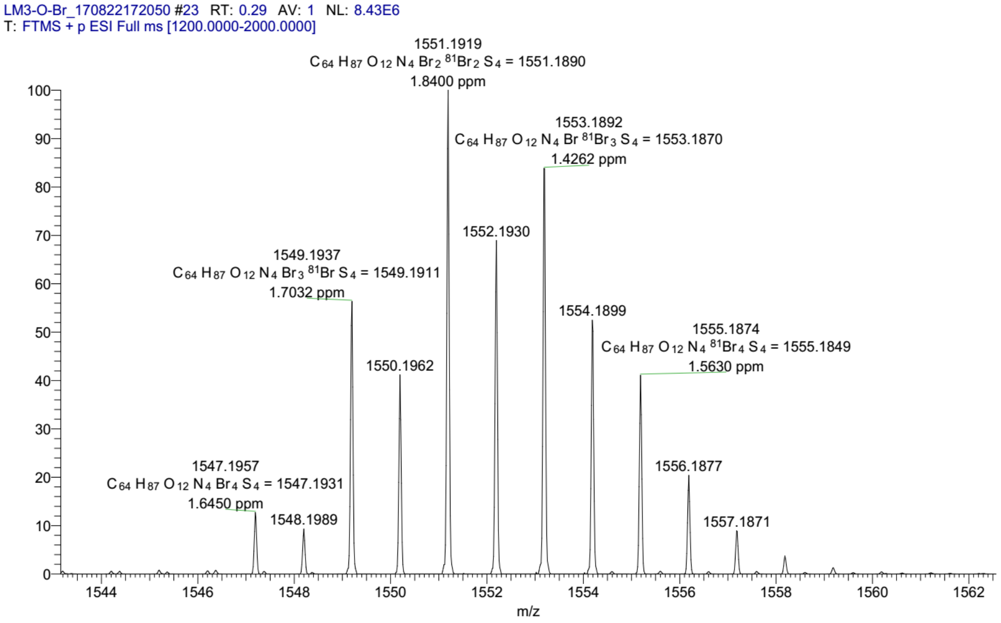

Supplementary Figure 27. HR MASS spectrum of **BGO6**.

Supplementary Table 1. Calculated first vertical S_0-S_1 excitation energies (E_{01}), first vertical S_1-S_0 emission energies (E_{10}), electronic configurations determined at the TD-ωB97XD*/6-31G (d) level of theory. [a]The optimally tuned range-separated parameters included in the functionals. [ex]Experimental data.

Molecules	ω*[a]	E_{01} (λ_{01}) ev (nm)	f_{01}	Electronic configuration	E_{10} (λ_{10}) ev (nm)	f_{10}	λ_{01}^{ex} (nm)	λ_{10}^{ex} (nm)
BGM6	0.1182	1.62(764)	0.32	HOMO → LUMO 99%	1.14(1092)	0.32	736	1047
BGP6	0.1182	1.59(780)	0.39	HOMO → LUMO 98%	1.13(1094)	0.36	736	1060
BGO6	0.1187	1.59(781)	0.39	HOMO → LUMO 98%	1.13(1093)	0.36	741	1060

Supplementary Table 2. Size-exclusion chromatography (SEC) analysis of the NIR-II molecular fluorophores.

Fluorophores	RV (mL)	M_n (Daltons)	M_w (Daltons)	M_w/ M_n
BGM6P	14.97	7191	7676	1.067
BGP6P	14.78	7523	8006	1.064
BGO6P	14.85	7486	7953	1.063

Supplementary Table 3. Pass percentage analysis of the fluorophores with different filters.

	Filtration	OD at peak	Pass percentage (%)
BGM6P	No filtration	3.91	100.00
	100k filtration	3.64	93.10
	50k filtration	0.98	25.10
	30k filtration	0.04	1.08
BGP6P	No filtration	3.83	100.00
	100k filtration	3.23	84.33
	50k filtration	0.36	9.40
	30k filtration	0.00	0.00
BGO6P	No filtration	3.77	100.00
	100k filtration	2.32	61.53
	50k filtration	0.14	3.66
	30k filtration	0.00	0.00

4 Reference

Bayly, C.I., Cieplak, P., Cornell, W., and Kollman, P.A. (1993). A well-behaved electrostatic potential based method using charge restraints for deriving atomic charges: the RESP model. *The Journal of Physical Chemistry* 97**,** 10269-10280. doi: 10.1021/j100142a004

Heyd, J., Scuseria, G.E., and Ernzerhof, M. (2003). Hybrid functionals based on a screened Coulomb potential. *Journal of Chemical Physics* 118**,** 8207. doi: 10.1063/1.1564060

Jorgensen, W.L., Chandrasekhar, J., Madura, J.D., Impey, R.W., and Klein, M.L. (1983). Comparison of simple potential functions for simulating liquid water. *The Journal of Chemical Physics* 79**,** 926-935. doi: 10.1063/1.445869

Lee, C., Yang, W., and Parr, R.G. (1988). Development of the Colle-Salvetti correlation-energy formula into a functional of the electron density. *Phys Rev B Condens Matter* 37**,** 785-789. doi: 10.1103/physrevb.37.785

Ma, H., Liu, C., Hu, Z., Yu, P., Zhu, X., Ma, R., Sun, Z., Zhang, C.-H., Sun, H., Zhu, S., and Liang, Y. (2020). Propylenedioxy Thiophene Donor to Achieve NIR-II Molecular Fluorophores with Enhanced Brightness. *Chemistry of Materials* 32**,** 2061-2069. doi: 10.1021/acs.chemmater.9b05159

Runge, E., and Gross, E.K.U. (1984). Density-Functional Theory for Time-Dependent Systems. *Physical Review Letters* 52**,** 997-1000. doi: 10.1103/PhysRevLett.52.997

Ryckaert, J.-P., Ciccotti, G., and Berendsen, H.J.C. (1977). Numerical integration of the cartesian equations of motion of a system with constraints: molecular dynamics of n-alkanes. *Journal of Computational Physics* 23**,** 327-341. doi: https://doi.org/10.1016/0021-9991(77)90098-5

Tomasi, J., Mennucci, B., and Cammi, R. (2005). Quantum Mechanical Continuum Solvation Models. *Chemical Reviews* 105**,** 2999-3094. doi: 10.1021/cr9904009

Wan, H., Ma, H., Zhu, S., Wang, F., Tian, Y., Ma, R., Yang, Q., Hu, Z., Zhu, T., Wang, W., Ma, Z., Zhang, M., Zhong, Y., Sun, H., Liang, Y., and Dai, H. (2018). Developing a Bright NIR-II Fluorophore with Fast Renal Excretion and Its Application in Molecular Imaging of Immune Checkpoint PD-L1. *Advanced Functional Materials* 28**,** 1804956. doi: https://doi.org/10.1002/adfm.201804956

Wang, J., Wolf, R.M., Caldwell, J.W., Kollman, P.A., and Case, D.A. (2004). Development and testing of a general amber force field. *Journal of Computational Chemistry* 25**,** 1157-1174. doi: https://doi.org/10.1002/jcc.20035

Yang, Q., Hu, Z., Zhu, S., Ma, R., Ma, H., Ma, Z., Wan, H., Zhu, T., Jiang, Z., Liu, W., Jiao, L., Sun, H., Liang, Y., and Dai, H. (2018). Donor Engineering for NIR-II Molecular Fluorophores with Enhanced Fluorescent Performance. *Journal of the American Chemical Society* 140**,** 1715-1724. doi: 10.1021/jacs.7b10334

Yang, Q., Ma, Z., Wang, H., Zhou, B., Zhu, S., Zhong, Y., Wang, J., Wan, H., Antaris, A., Ma, R., Zhang, X., Yang, J., Zhang, X., Sun, H., Liu, W., Liang, Y., and Dai, H. (2017). Rational Design of Molecular Fluorophores for Biological Imaging in the NIR-II Window. *Advanced Materials* 29**,** 1605497. doi: https://doi.org/10.1002/adma.201605497
